# Misfolded protein oligomers: mechanisms of formation, cytotoxic effects, and pharmacological approaches against protein misfolding diseases

**DOI:** 10.1186/s13024-023-00651-2

**Published:** 2024-02-20

**Authors:** Dillon J. Rinauro, Fabrizio Chiti, Michele Vendruscolo, Ryan Limbocker

**Affiliations:** 1 Centre for Misfolding Diseases, Yusuf Hamied Department of Chemistry, University of Cambridge, Cambridge, CB2 1EW UK; 2 Section of Biochemistry, Department of Experimental and Clinical Biomedical Sciences, University of Florence, 50134 Florence, Italy; 3 Department of Chemistry and Life Science, United States Military Academy, West Point, NY 10996 USA

**Keywords:** Aggregation kinetics, Secondary nucleation, Protofibrils, Lecanemab, Cellular interactions, Amyloid toxicity, Drug discovery, Diagnostics, Therapeutics, Fibril fragmentation, Biophysics

## Abstract

The conversion of native peptides and proteins into amyloid aggregates is a hallmark of over 50 human disorders, including Alzheimer’s and Parkinson’s diseases. Increasing evidence implicates misfolded protein oligomers produced during the amyloid formation process as the primary cytotoxic agents in many of these devastating conditions. In this review, we analyze the processes by which oligomers are formed, their structures, physicochemical properties, population dynamics, and the mechanisms of their cytotoxicity. We then focus on drug discovery strategies that target the formation of oligomers and their ability to disrupt cell physiology and trigger degenerative processes.

## Background

It has been known for many years that numerous normally soluble proteins can misfold and self-assemble into amyloid fibrils [1–5]. The architecture of these aberrant aggregates is highly organized, with a characteristic cross-β core formed by β-strands arranged perpendicular to the main axis of the fibril, creating an extensive network of hydrogen bonds that confer high stability to the amyloid state [1–4, 6]. Amyloid fibrils are associated with a variety of human diseases involving either the central nervous system (neuropathic conditions), a multiplicity of tissues and organs other than the brain (non-neuropathic systemic amyloidoses), or a specific organ (non-neuropathic localized amyloidoses) [1]. Individual amyloid-associated diseases (Table 1) are generally characterized by the loss of native function for specific peptides or proteins, and these biomolecules can form aberrant and destructive aggregates [1, 7]. Collectively, these conditions affect dozens of millions of people worldwide [1]. To cite a few examples, the deposition in the brain of the tau protein into intracellular neurofibrillary tangles [8, 9] and the amyloid-β peptide (Aβ) into extracellular plaques [10, 11] is associated with Alzheimer’s disease (AD). Parkinson’s disease (PD) is characterized by the aggregation of ⍺-synuclein into Lewy Bodies of dopaminergic neurons [12, 13], and type II diabetes (T2D) by the self-assembly of islet amyloid polypeptide (IAPP, also known as amylin) in the islets of Langerhans in the pancreas [14, 15]. Consequently, the characterization of the various species that are formed within each aggregation reaction, and the study of the mechanisms by which they contribute to cellular dysfunction and death, would help reveal the molecular origins of protein misfolding diseases and provide insights into possible therapeutic and diagnostic methods to combat these conditions.

**Table 1:** Non-exhaustive list of protein misfolding diseases with their associated peptides or proteins, and in vitro and ex vivo structures of corresponding amyloid fibrils. Putative mechanisms of aggregation and toxicity are also indicated

Disease	Proteins	Length	Prospective most toxic species	Dominant aggregation mechanism	In vitro amyloid structure	Ex vivo amyloid structure
Alzheimer’s disease (AD)	Amyloid-β (Aβ) Tau (3R + 4R)	40 or 42 352–441	Aβ: Oligomers [3, 16, 17] Tau: Oligomers [18–20]	Secondary nucleation [21, 22] Secondary nucleation [23]	Aβ_40_ [24] Aβ_42_ [25] Tau [26]	Aβ_40_ [27, 28] Aβ_42_ [5] Tau [29, 30]
Parkinson’s disease (PD)	⍺-synuclein	140	Oligomers [31–33]	Condition-dependent [34], including lipid-induced aggregation [35]	[36–39]	[40–42]
Dementia with Lewy Bodies (DLB)	⍺-synuclein	140	Pre-synaptic aggregates [43] & oligomers [44, 45]	Not yet known, secondary pathways [46]	-	[40, 47]
PD dementia (PDD)	⍺-synuclein	140	Not yet known, possibly oligomers [48]	-	-	[40]
Multiple system atrophy (MSA)	⍺-synuclein	140	Oligomers [49, 50]	-	-	[42, 51]
Huntington’s disease (HD)	Huntingtin	variable	Not yet known, possibly oligomers [52–54]	Possibly stochastic nucleation [55]	[56]	-
Chronic traumatic encephalopathy (CTE)	Tau (3R + 4R)	352–441	Not yet known	Not yet known, possibly secondary pathways [57]	[26]	[58]
Pick’s disease (PiD)	Tau (3R)	352–410	Not yet known	Not yet known, possibly secondary pathways [59]	-	[60]
Corticobasal degeneration (CBD)	Tau (4R)	383–441	Not yet known	Not yet known, possibly secondary pathway [61]	-	[62]
Progressive supranuclear palsy (PSP)	Tau (4R)	383–441	Not yet known	-	-	[4]
Argyrophilic grain disease (AGD)	Tau (4R)	383–441	Not yet known	Not yet known, possibly secondary pathways [63]	-	[4]
Globular glial tauopathy (GGT)	Tau (4R)	383–441	Not yet known	Not yet known, possibly secondary pathways [64]	-	[4]
Spongiform encephalopathies	Prion protein (PrP)	208	PrP^Sc^ [65], possibly oligomers [66, 67]	Fragmentation [68]	[69–72]	Mouse PrP [73, 74] RML prion [75] Scrapie [70] Bovine serum encephalopathy [76]
Type II diabetes	Amylin (IAPP)	37	Oligomers [77, 78]	Secondary nucleation [79]	[80, 81]	[82]
Cataracts	Crystallins	175	Aggregates of damaged proteins [83, 84]	-	-	-
AA amyloidosis	Serum amyloid A	104	Fibrils [85] and oligomers [86]	Secondary pathways [87]	[85, 88]	[85, 88]
Transthyretin amyloidosis	ATTRwt Val30Met ATTR	127	Various species [89–91]	-	-	[92, 93]
Ig-related amyloidosis (AL)	Immunoglobulin light chain	Proteolytic fragments	Fibrils [94] and oligomers [95]	Not yet known, seed-competent [96]	-	[97, 98]

The fibrillar species of amyloidogenic proteins were initially thought to be the most toxic aggregate forms in many protein misfolding diseases. However, increasing evidence has shown that smaller, intermediate and metastable soluble aggregates, known as misfolded protein oligomers, are in many cases more toxic than their mature fibrillar counterparts [18, 19, 77, 99–104]. In other protein misfolding diseases, amyloid fibrils are cytotoxic either by sequestering functional proteins (loss-of-function) or by directly damaging the cells and tissues where they form (toxic gain-of-function) [1, 100, 105, 106]. Moreover, once formed, amyloid fibrils can, in certain amyloid systems, establish a positive feedback loop that further promotes the proliferation of more oligomers in a multiplicative manner through surface-catalyzed secondary nucleation [21, 107]. Furthermore, amyloid fibrils can act as reservoirs of oligomers that can detach from the fibril ends [108]. Mature fibrillar aggregates also remain main pathological biomarkers and histological hallmarks [109–112], while oligomers may be a crucial target for effective drug screening programs [16, 101, 113, 114].

Because of the elusive nature of misfolded protein oligomers, they can vary in their characteristics, including differences in their size and hydrophobicity, as well as their degree of metastability [1, 115, 116]. The detection of oligomers ex vivo for diagnostic purposes thus presents challenges, particularly due to their heterogeneous structure, transient nature, and low concentrations [115]. Aβ oligomers have been detected in AD brains using conformation-sensitive antibodies specific for well-defined oligomers with low reactivity towards monomers, fibrils and other oligomer types [115, 117–122]. Aβ oligomers have also been detected in peripheral fluids, including the cerebrospinal fluid (CSF), where they are present in the circa attomolar to picomolar concentration range [115, 123, 124], and tau oligomers have been found in the CSF at approximately femtomolar concentrations [125].

Despite these challenges, progress has been made to reinforce our understanding of misfolded protein oligomers on several fronts, as we will describe in this review article. We consider here these oligomers with respect to their biophysical characterization and population dynamics, quantification techniques, the processes by which they form, and the consequences of their dysregulated presence. We then conclude by summarizing recent developments in drug discovery against oligomers.

## The amyloid state of proteins

**Fig. 1:**
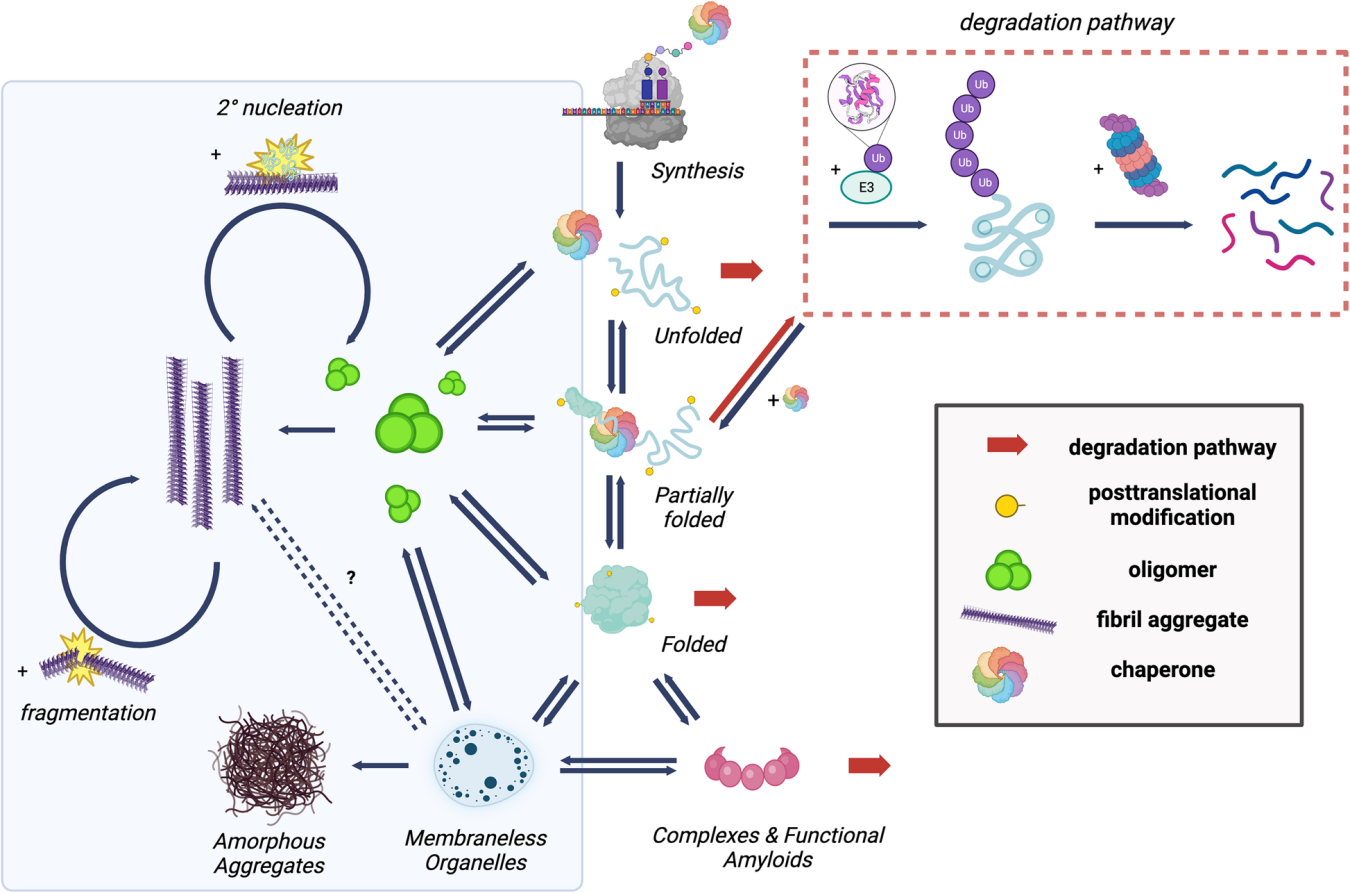
Proteins interconvert between different conformational states in the cell. After its biosynthesis by the ribosome, a protein may fold into its native state and be trafficked to its correct cellular location, assemble into a functional complex, condense into membraneless organelles, or misfold and aggregate. These processes are regulated by the proteostasis network (PN) [1, 140]. Accessing the amyloid state is a process that typically involves the conversion of monomeric proteins into oligomers and ultimately, highly ordered, rigid cross-β sheet fibrillar structures [1]. In addition to being associated with disease, amyloid fibrils can also be functional, and for this reason they have applications in material sciences, biomedical engineering, and drug discovery [1, 2, 141]. Created with biorender.com

Many proteins in their physiological states are frequently expressed at levels close to their solubility limits [126, 127]. These proteins are thermodynamically metastable in their native states and over time tend to convert into aggregates [128] (Fig. 1). The amyloid state is characterized by the presence of fibrillar aggregates consisting of a number of β-sheet structures running along the fibril axis [129, 130]; a fibrillar morphology with characteristic cross-β structure and signature tinctorial properties, including binding of the dyes thioflavin-T and Congo red, are commonly accepted as key hallmarks of the amyloid state [1]. It is accessible independently of the sequence, structure and function of the precursor native proteins and is now recognized to often be the most stable state of a protein, even more stable than the native state, at the high protein concentrations present in the cellular environment [131, 132]. Breakthrough developments in solid-state nuclear magnetic resonance (ssNMR) spectroscopy and then cryogenic electron microscopy (cryo-EM), particularly the increased sensitivity of instruments [133] and the advancement of analytical software [134], have facilitated the determination of high-resolution structures (approaching the 2 Å limit) of brain-derived amyloid fibrils of disease-associated proteins and peptides. Filamentous structures have been solved for many amyloidogenic biomolecules, including for tau [135], TDP-43 [136], ⍺-synuclein [40], and amyloid-β [5], although those of TDP-43 do not exhibit cross-β structure with the 10–11 Å spacing and binding to amyloid diagnostic dyes [136]. Remarkably, it has been shown that the tau protein can self-assemble into a range of different amyloid structures, known as polymorphs, in a pathology-dependent manner, including in AD, Pick’s disease (PiD), chronic traumatic encephalopathy (CTE), corticobasal degeneration (CBD), and progressive supranuclear palsy (PSP) [4, 135] (Table 1), and the same is true for amyloid fibrils formed from other proteins [137]. Polymorphism has also been observed for Aβ fibrils in AD, with different polymorphs in different patients [5, 138], and in familial and sporadic forms [139].

We also note that in some cases amyloid species can be functional, and be formed both intra- and extra-cellularly in diverse organisms ranging from bacteria to mammals [142–144]. For example, functional amyloids serve roles in curli formation in *E. coli*, in the control of nitrogen catabolism in yeast, and as scaffolds that promote melanin synthesis in human melanocytes [142, 143]. Unlike pathological amyloid fibrils, functional amyloids form under controlled conditions and seem to have evolved to avoid secondary pathways, possibly in order to eliminate autocatalytic processes that would be difficult to regulate [145]. From a materials chemistry perspective, functional synthetic amyloids have been leveraged for a variety of biotechnological applications, such as silk micrococoons for antibody drug delivery [146], and amyloid-coated purification systems to reduce heavy metal concentrations in contaminated water sources [147]. Although predominantly studied for their pathogenicity, fibrillar species from protein misfolding diseases have also demonstrated a capacity to catalyze chemical reactions. For example, it has been shown recently that various amyloid fibrils can also catalyze chemical reactions, including the hydrolysis of para-nitrophenyl acetate and dephosphorylation of para-nitrophenyl-orthophosphate induced by ⍺-synuclein fibrils [148] and the degradation of specific neurotransmitters induced by Aβ fibrils [149].

## Structure and mechanism of oligomer formation

The conversion of proteins from their soluble native state to amyloid fibrils is a complex process that involves a number of intermediate states. We will refer to the intermediates that are multimeric but small enough to remain soluble as misfolded protein oligomers. A great assortment of oligomers has been described for the widely studied Aβ_40_/Aβ_42_ system and a wide range of names have been given them, such as spherical and chain-like protofibrils, paranuclei, pentamers, globulomers, amylospheroids, SDS-stable dimers/trimers, Aβ-derived diffusible ligands (ADDLs), prefibrillar and fibrillar oligomers, and spherical amyloid intermediates. [101, 118, 120, 150–159]. Different oligomeric species have also been described for ⍺-synuclein, including type A, type A*, type B and type B* oligomers, amongst many other forms [33, 108, 160–163].

Comparison between structural characteristics of the various oligomers indicates that the β-sheet content generally increases with molecular weight, suggesting that an increase in oligomer size stabilizes their β-sheet structure. Such structure has generally been shown to involve both anti-parallel [157, 161, 164] and parallel but out-of-register strands [165], unlike fibrils where β-strands are generally parallel and in-register. When various oligomers of Aβ or ⍺-synuclein appear sequentially with time during an aggregation process, the first species are unstructured, and the species containing β-sheet structure appear later [154, 159, 160, 165, 166]. The aggregation of globular proteins recapitulates many of these characteristics if the process takes place under conditions promoting their unfolding [167, 168]. However, when it is initiated under native conditions, it often leads to early aggregates where the individual monomers populate native-like states, which later convert into β-sheet containing protofibrils/fibrils [169, 170].

The largest oligomers with the highest β-sheet content, such as the fibrillar oligomers, annular protofibrils, chain-like protofibrils and amylospheroids for Aβ and type B or B* oligomers for ⍺-synuclein, represent off-pathway species that need to reassemble at least partially before forming amyloid fibrils [164, 171, 172]. These species are large and have antiparallel or parallel but out-of-register β-sheet arrangements that needs to be substantially reorganized to form the parallel in-register cross-β structure of the fibrils. Their off-pathway nature is also shown by dedicated kinetic tests [164, 171, 173]. On-pathway oligomers are more difficult to detect and isolate because they generally convert into other oligomers or fibrils. Indeed, one important class of oligomers include nuclei of fibril formation, which can be identified kinetically, as described in the next section.

## Kinetic mechanisms of amyloid fibril formation to reveal oligomeric nuclei

### Macroscopic measurements

**Fig. 2:**
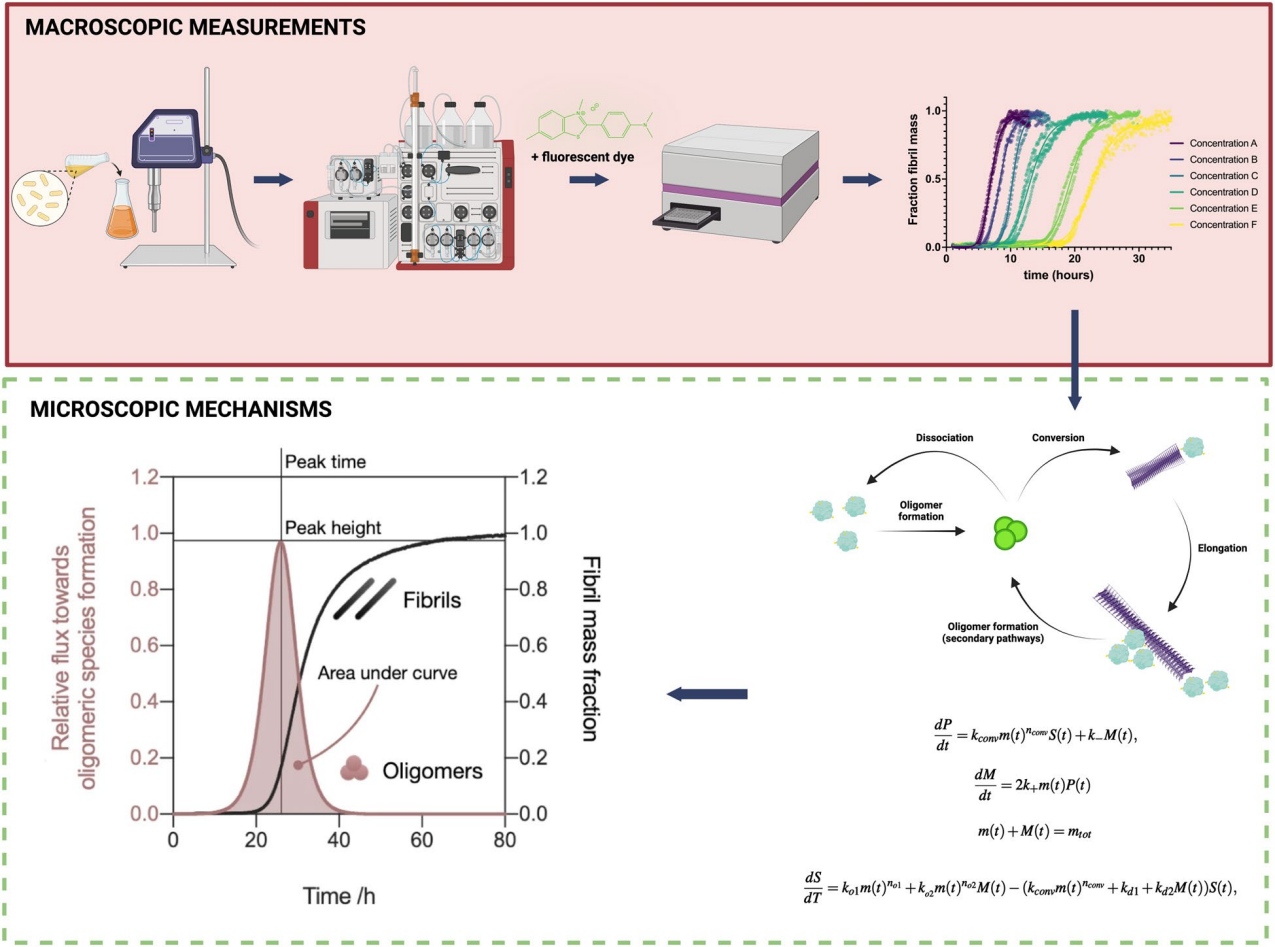
From macroscopic measurements to microscopic mechanisms of protein aggregation. In a typical in vitro aggregation experiment, recombinant proteins are purified using a number of procedures, including fast protein liquid chromatography. Samples containing purified proteins are aliquoted with an amyloid-binding fluorophore in a low-bind multiwell plate. A plate reader tracks the time-dependent evolution of the overall fibril mass, and kinetic traces can be subsequently analyzed using chemical kinetics to resolve the mechanism of aggregation, as well as the effects of additive species, such as aggregation inhibitors. The reactive flux towards oligomeric species can be calculated using this approach [179]. Relative flux graphic reprinted from Staats et.al [179]. Created with biorender.com

Quantitative kinetic analysis of amyloid fibril formation makes it possible to gain insight into the mechanism of misfolded protein oligomer generation that are on-pathway to fibril formation, as well as their population dynamics [174]. Chemical kinetics enables the establishment of models to describe the conversion of monomeric proteins into fibrillar products by breaking the process down into a series of elementary steps governed by rate laws [175]. Fluorescent dyes are commonly used to monitor fibril formation, as they exhibit a substantial increase in quantum yield upon their interaction with β-sheet rich structures (Fig. 2) [176, 177]. Consequently, the binding of these dyes to amyloid fibrils induces a large fluorescence emission increase that, over time, manifests as a classic sigmoidal curve in in vitro aggregation assays. Macroscopically, this sigmoidal curve can be thought of as having three major phases: (1) a lag phase where aggregation is already under way, but the amount of fibrillar structures is too low to be detectable, (2) a growth phase dominated by secondary processes (i.e. microscopic steps that depend on the presence of fibrils), and (3) a plateau phase that begins when the concentration of the monomers remaining in solution becomes rate limiting [178]. However, because of their ability to recognize molecular grooves of a fibrillar surface, the dyes are not ideally suited to quantify the heterogeneous species formed in the early stages of the aggregation process, including oligomers and protofibrils. Biophysical methods can instead be used, such as dynamic light scattering (DLS) or microfluidic free-flow electrophoresis [115], amongst others discussed later in this review.

To connect these macroscopic observables with the microscopic processes that contribute to the overall reaction, one can use the formalism of chemical kinetics (Table 2). In this approach, the elementary steps underlying the aggregation process are described by a system of differential equations, known as a master equation. This equation defines the time dependence of the populations of the intermediate species produced during the reaction, which cannot be readily measured, from the knowledge of the time dependence of the populations of the reactants and products, which can instead be measured [180]. The steps accounted for within the master equation approach for amyloid systems can be classified into two groups: those that affect the aggregate mass (growth), such as fibril elongation and monomer dissociation, and those that contribute to the total number of aggregates, such as primary nucleation, secondary nucleation, and fibril fragmentation.

**Table 2:** Time evolution of the fibril number concentration (P), fibril mass concentration (M) and oligomer mass concentration (S) for the microscopic processes involved in amyloid formation [181]. m(t), time-dependent monomer concentration; M(t), time-dependent fibril mass concentration; P(t), time-dependent fibril number concentration; S(t), time-dependent oligomer mass concentration; k_n_, primary nucleation rate constant; k_2_, secondary nucleation rate constant; k_+_, elongation rate constant; k_-_, fragmentation rate constant; k_d_, oligomer dissociation rate constant; k_o1_, primary oligomer association rate constant; k_o2_, secondary oligomer association rate constant; k_conv_, oligomer conversion rate constant; k_d2_, fibril-mediated oligomer dissociation rate constant; n_c_, reaction order of primary nucleation; n_2_, reaction order of secondary nucleation; n_conv_, reaction order of oligomer conversion; n_o1_, reaction order of primary oligomer association; n_o2_, reaction order of secondary oligomer association; K_E_, Michaelis–Menten constant for saturating elongation (monomer concentration at which the rate of elongation is half the maximal velocity, V_max_); K_M_ (Michaelis–Menten constant for saturating secondary nucleation (monomer concentration at which the rate of secondary nucleation is half the maximal velocity, V_max_)

Molecular process	Time evolution model	Illustration of the model
Primary nucleation	$\frac{dP}{dt}={k}_{n}m{\left(t\right)}^{{n}_{c}}$	
Heterogeneous primary nucleation	*varies*	
Elongation	$\frac{dM}{dt}=2{k}_{+}m\left(t\right)P\left(t\right)$	
Saturating elongation	$\frac{dM}{dt}=\frac{2{k}_{+}m\left(t\right)P\left(t\right)}{1+\frac{m\left(t\right)}{{K}_{E}}}$	
Fragmentation	$\frac{dP}{dt}={k}_{-}M\left(t\right)$	
Secondary nucleation	$\frac{dP}{dt}={k}_{2}m{\left(t\right)}^{{n}_{2}}M\left(t\right)$	
Multistep secondary nucleation	$\frac{dP}{dt}=\frac{{k}_{2}m{\left(t\right)}^{{n}_{2}}M\left(t\right)}{{\left(1+\frac{m\left(t\right)}{{K}_{M}}\right)}^{{n}_{2}}}$	
Primary association of oligomers	$\frac{dS}{dt}={k}_{o1}m{\left(t\right)}^{{n}_{o1}}$	
Primary dissociation of oligomers	$\frac{dS}{dt}={k}_{d}S\left(t\right)$	
Oligomer conversion	$\frac{dS}{dt}={k}_{conv}m{\left(t\right)}^{{n}_{conv}}S\left(t\right)$	
Secondary association of oligomers	$\frac{dS}{dt}={k}_{o2}m{\left(t\right)}^{{n}_{o2}}M\left(t\right)$	
Fibril-mediated oligomer dissociation	$\frac{dS}{dt}={k}_{d2}S\left(t\right)M\left(t\right)$	

**Fig. 3:**
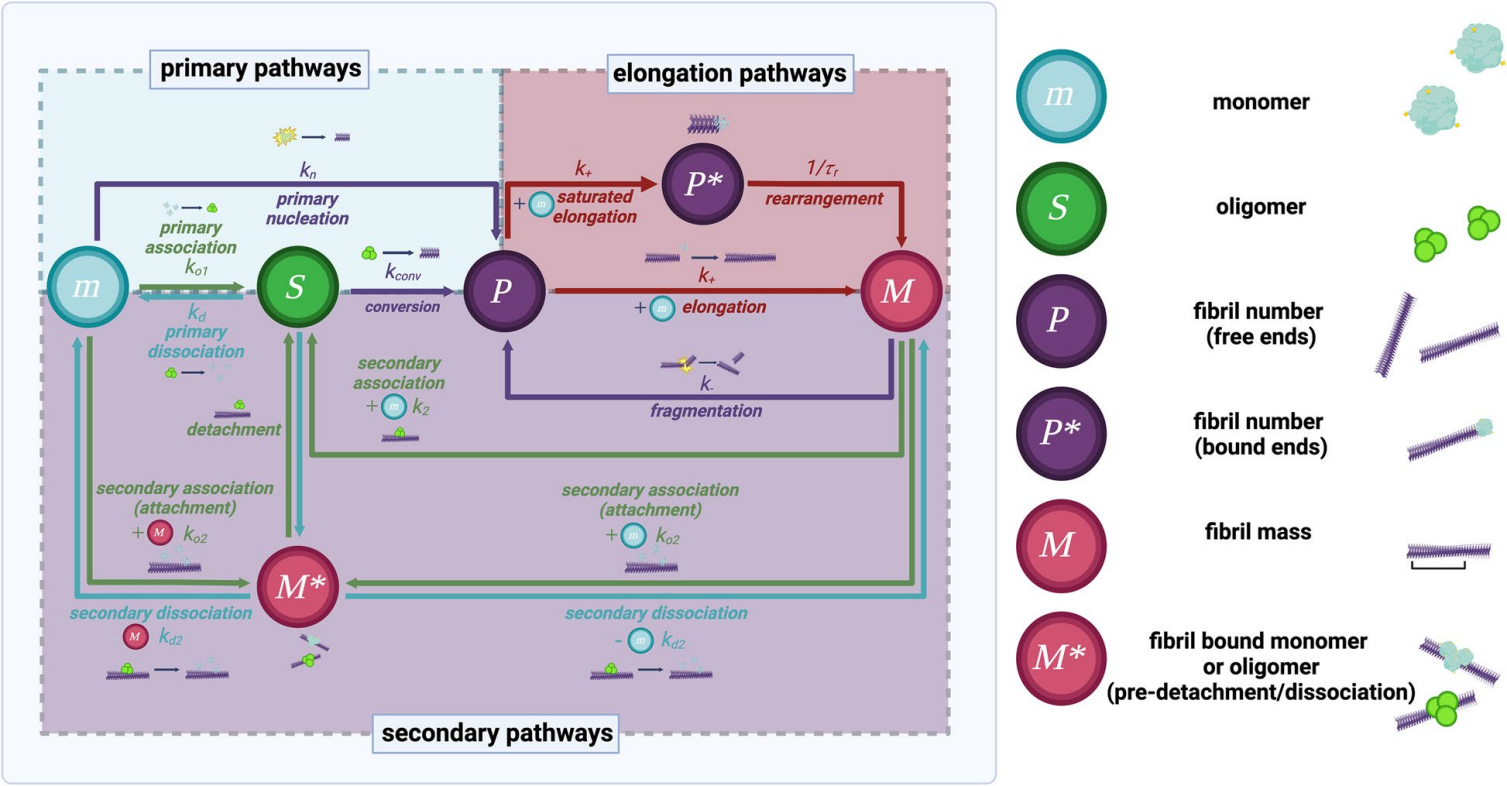
Petri net representation of the reaction network that models an aggregation reaction. The monomer mass concentration (m), fibril mass concentration (M), fibril number concentration (P) and oligomer mass concentration (S), as well as the rate constants of their interconversions (see Table 2 for definitions), are indicated. M* represents monomer bound to fibril prior to its conversion or detachment, and P* represents a multistep elongation process including association and rearrangement. These processes fall into three categories: growth processes (elongation pathways), primary pathways, and secondary pathways (i.e. those that require the presence of fibrils). Note that no fibrillar mass is lost due to secondary nucleation or fragmentation, unless non-fibrillar oligomers are capable of detaching from fibril ends. Pathways shown in red and purple increase the relative fibrillar mass and number, respectively. In green are the pathways considered to be 'pro-oligomer,' meaning they lead to a net increase in the oligomer population. Conversely, pathways shown in blue represent microscopic processes that lead to oligomer dissociation. Adapted from Meisl et.al [185], to include the reactive flux towards and away from oligomers. Created with biorender.com

**Fig. 4:**
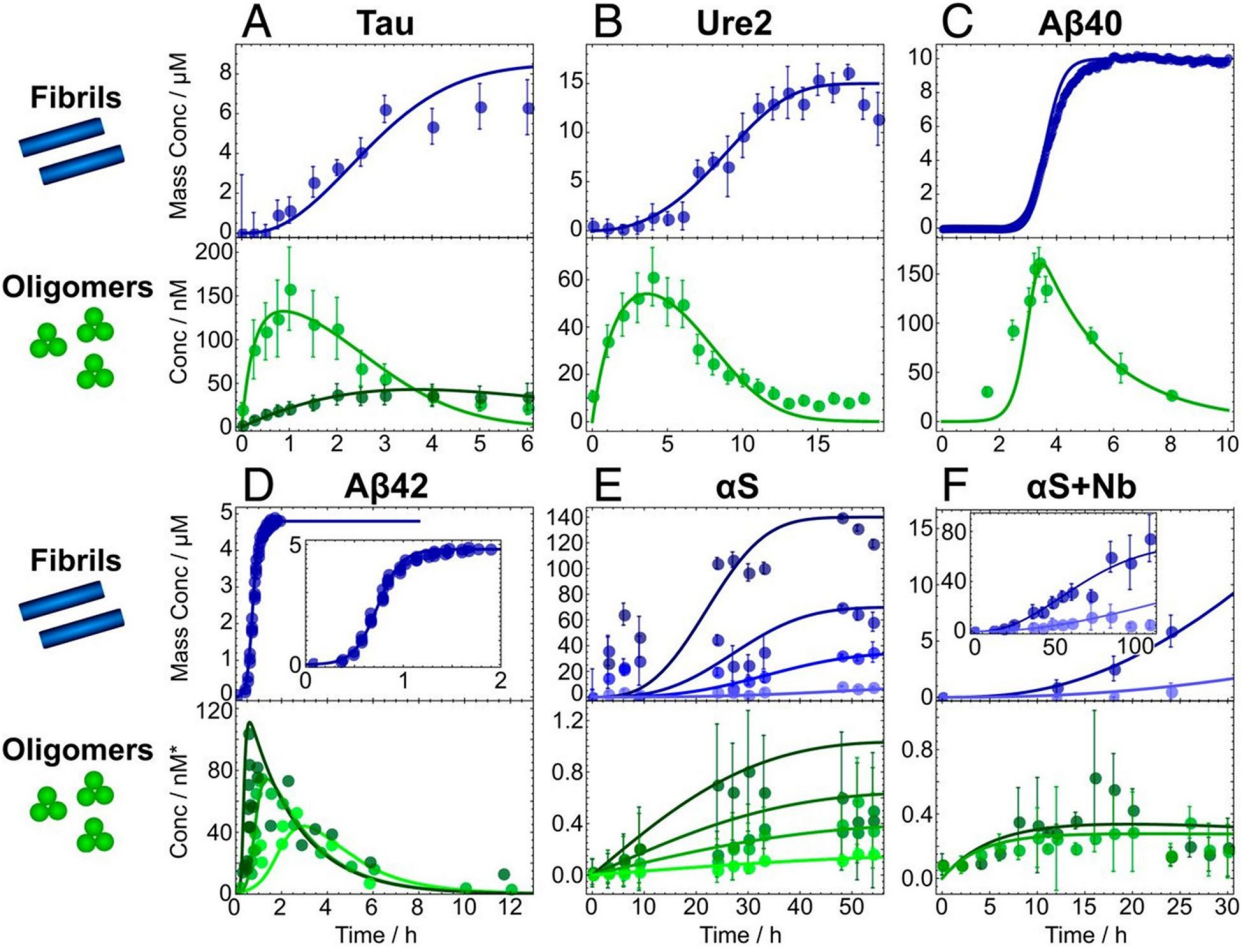
Global simultaneous fits for fibril mass concentration using amyloid-binding dyes and soluble oligomer concentration. Fibril (top panels) and oligomer (bottom panels) concentrations were determined using amyloid-binding dyes and single molecule biophysical techniques, respectively. Fitting parameters are summarized herein and described in detail in ref. [186]. This study shows that it is possible to monitor oligomer dynamics from macroscopic amyloid-dye binding experiments. Reprinted from Dear et. al [186]

In practice, a series of aggregation assays are conducted with different initial conditions to probe specific microscopic mechanisms [175, 181]. Specifically, the addition of low concentrations of fibrillar seeds at the start of the aggregation reaction can bypass primary nucleation, while the addition of high concentrations of seeds bypasses both primary and secondary nucleation, thus assessing elongation. The combination of unseeded and seeded aggregation assays can offer sufficient constraints to enable the determination of the microscopic rate constants by a global fit of the kinetic data [180, 182–184]. The web platform Amylofit, which is freely available (https://amylofit.com/), has been developed to facilitate this type of analysis [181], and it can solve molecular mechanisms and kinetic parameters with and without additives like small molecules. The differential equations for key elementary steps in the amyloid pathway are summarized in Table 2, and the microscopic steps relevant to oligomer formation (Fig. 3) are analyzed in the following sections. Examples of individual kinetic traces from several different protein systems analyzed with this analytical procedure and single-molecule biophysical experiments are also shown in Fig. 4.

### Fibril elongation

Elongation is a microscopic process in which a monomer is added to a fibril end. The rate at which this process takes place, specified by the rate constant k_+_, is highly dependent upon conditions such as temperature, pH, and ionic strength [187]. Generally, fibril elongation sees the adsorption of monomer to the ends of a growing fibril, followed by a rapid conversion step. However, at sufficiently high concentrations of monomers, fibril elongation can be saturated, suggesting a two-step process [188].

### Primary nucleation

In this process, individual monomers self-associate into small, disordered oligomers. The majority of the oligomers produced in this way dissociate back into monomers [174], though in some cases they can persist long enough to convert into ordered oligomers, which are effectively fibril fragments and can act as seed for fibril growth [174]. The presence of surfaces, for example other proteins or lipid membranes, can, in certain instances, serve as a catalyst that induces heterogeneous primary nucleation [189, 190]. It is difficult to observe individual primary nuclei in a macroscopic sample due to their low concentration, transient nature, and a lack of molecular probes that bind them with specificity. The use of microfluidic techniques has, however, enabled their visualization, thus providing novel insight into the role of system size on primary nucleation events and the propagative nature of the amyloid cascade [191].

### Secondary nucleation

The process by which new aggregates form by a monomer adsorbing onto and nucleating at the surface of an existing fibril is known as secondary nucleation [192]. It has been observed crystalline systems [193] and a range of protein deposition reactions, including for sickle-cell hemoglobin [194], IAPP [80, 195], insulin [196], Aβ_42_ [21], ⍺-synuclein [35, 197], and tau [23], with increasing evidence implicating its key role in disease and pathology. Secondary nucleation in protein aggregation has been directly visualized using direct stochastic optical reconstruction microscopy (dSTORM) [198] and total internal reflection fluorescence (TIRF) microscopy [199]. Furthermore, the formation of oligomers is greatest when both monomers and fibrils are involved in an aggregation reaction, compared to any other molecular process in the amyloid network. Thus, secondary nucleation, markedly more so than other microscopic steps, can be often implicated as a major source of toxic misfolded protein oligomers (Fig. 3) [200].

### Fragmentation

Concurrent with or independent of secondary nucleation depending on solution conditions, fragmentation is another secondary pathway which may be particularly relevant, such as for the aggregation of insulin [180], β-lactoglobulin [201, 202], and the prion glycoprotein [203]. In a process dominated by fibril fragmentation, a fibril breaks into shorter fibril fragments, leading to a proliferation of fibril ends and therefore an exponential increase in fibril mass in the presence of fibril elongation. Fibril fragmentation may result from thermal fluctuations and mechanical stress [204], or regulatory processes, such as by molecular chaperones [205]. In the real-time quaking-induced conversion (RT-QuIC) assay, monomeric substrate can be seeded by pathogenic fibrils present in a diluted brain homogenate sample alongside shaking to induce fragmentation, including for 3R [59, 206] and 4R [61] tauopathies, and synucleinopathies [207]. Like secondary nucleation, fragmentation can enhance the overall cytotoxicity within the amyloid cascade [208]. Exacerbated cytotoxicity may stem from the ability for oligomers to detach from fibril ends [108], the exponential growth of fibril ends for monomer adsorption, or enhanced cellular uptake of, on average, shorter fibrils [209–211].

### Oligomer dynamics

Recent advances in experimental methods to detect and quantify oligomers [115] (see below) have facilitated the development of kinetic models that explicitly include the formation and disruption of oligomers, including ones that are both on- and off-pathway (Table 2). This inclusion of oligomer dynamics in the rate equations for protein aggregation, and their subsequent fitting to data using amyloid-binding dyes that monitor for fibril formation, now enables the detailed description of the kinetics of oligomer populations formed during aggregation reactions for multiple protein systems [174, 186, 212].

As done with amyloid assembly kinetics, the evolution of oligomeric species can be broken down into a series of microscopic elementary steps: monomer association into oligomers, oligomer dissociation into monomers, oligomer conversion into oligomers competent for fibril elongation (fibrillar oligomers), and elongation of fibrillar oligomers to fibrils (Fig. 3**, **Table 2) [186]. Non-fibrillar (i.e. non-converted) oligomers can form via primary nucleation, which is a fibril-independent pathway, or via fibril-dependent secondary mechanisms (Table 2). Upon formation, oligomers can be depleted by either their conversion into elongation-competent fibrillar species, or their dissociation back into monomers (Table 2). Because of the reversibility of secondary nucleation, it is also possible that oligomers dissociate back to monomers upon interaction with the fibril surface. This is included in Fig. 3 and Table 2 as secondary dissociation or fibril-mediated oligomer dissociation. Each of these steps contributes to the oligomer population dynamics for a given amyloid system, which can be summarized by four main parameters that differ considerably from one amyloid system to another. These paramters are: persistence, productivity, abundance, and peak half-time.

The first of these, oligomer persistence, measures the decay, whether through dissociation or conversion, of the oligomer population upon reaching the peak concentration. It is governed by the average lifetime of the oligomers. Revisiting available in vitro kinetics data [21, 22, 213, 214] with a mechanistic model accounting for oligomeric reactions revealed that the intermediate species have different lifetimes ranging from a few minutes (PrP) to hundreds of hours (⍺-synuclein) under their corresponding conditions of analysis. 

Another parameter to describe the dynamics of oligomers, the kinetic productivity, measures the tendency of oligomers to convert into fibrillar nuclei instead of dissociating. Like persistance, the productivity of oligomers varies substantially between the various amyloid systems studied so far. From tau to α-synuclein, oligomer productivity can vary by over four orders of magnitude, reaching values up to approximately 23% [114]. A higher rate of productivity effectively translates to a reduction in the concentration of oligomers at any given time. 

Abundance, the maximum concentration of oligomers that can be estimated theoretically, is determined by the maximal rate of oligomer formation relative to the maximal rate of depletion. These rates are determined by the rates of primary nucleation and/or secondary nucleation, as applicable [114, 186]. This parameter is generally less variable between systems compared to the differences in productivity and half time, with predicted values as low as 0.3% for Ure2 and as high as 8% for ⍺-synuclein and Aβ42 [186]. 

Finally, the peak time refers to the timepoint in the experiment when oligomer concentration is maximal and is set by the characteristic rate of aggregation and initial monomer concentration, and it varies by approximately one order of magnitude for different proteins.

**Fig. 5:**
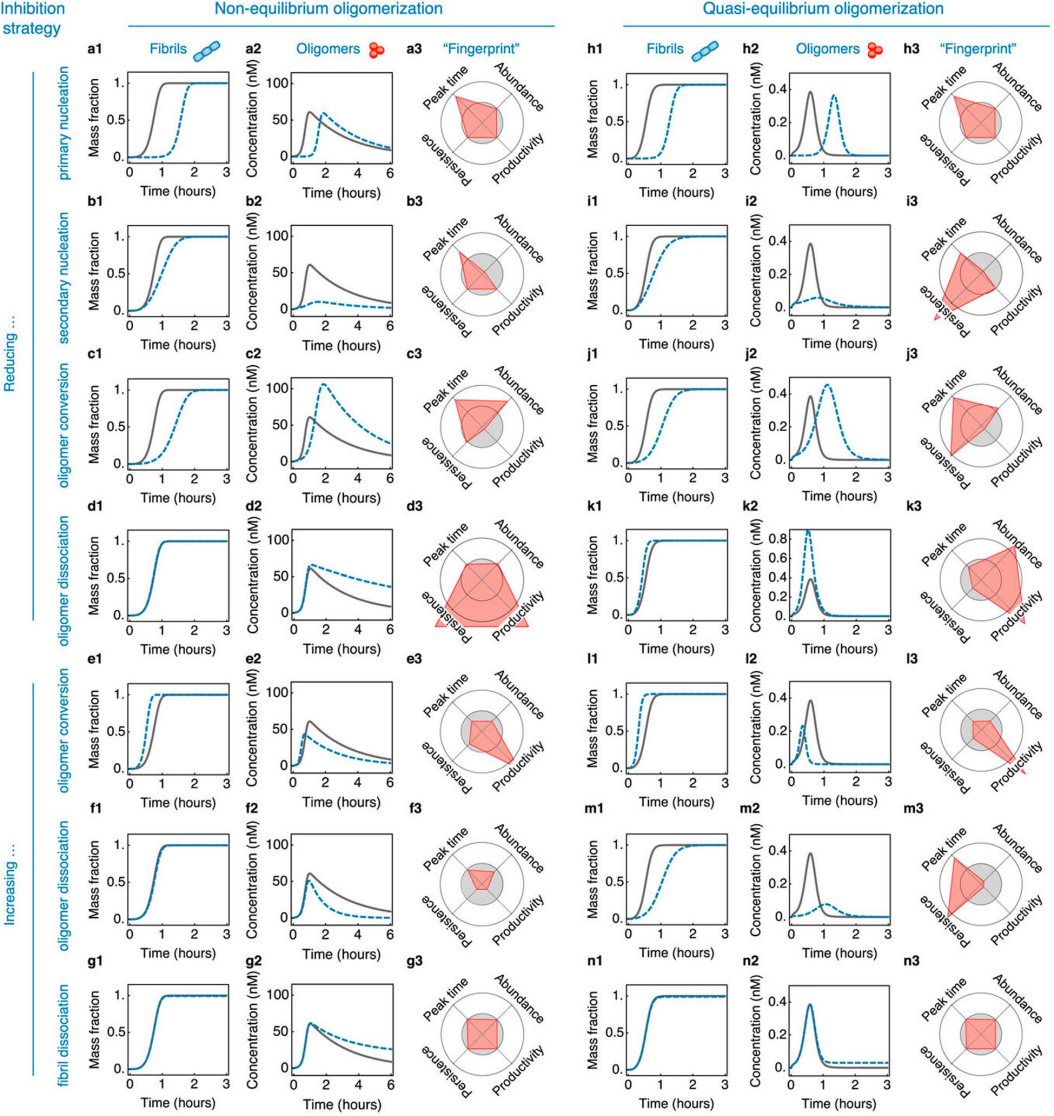
Simulated effects on the time evolution of fibril and oligomer populations upon addition of compounds that inhibit specific microscopic steps in the aggregation reaction of a given protein. Reprinted from Michaels et. al [114], with the permission of AIP Publishing

For systems dominated by secondary nucleation, these four experimentally observable parameters, peak time, productivity, persistence, and abundance, can be mathematically represented (Table 3). Consequently, these metrics facilitate the investigation of the various effects of potential therapeutics or additive agents on targeting one or more microscopic steps (Fig. 5).

**Table 3:** Analytical expressions for various descriptors of oligomer population dynamics [114]. ρ_o1_ and ρ_o2_ represent the rates of oligomer formation via primary and secondary nucleation, respectively, ρ_+_ represents the rate for fibril elongation, ρ_c_ represents the rate of oligomer conversion, and ρ_e_ represents the combined rates of oligomer conversion and dissociation

Descriptor	Non-equilibrium oligomerisation	Quasi-equilibrium oligomerisation
***Secondary nucleation-dominated***		
Peak time	${\tau }_{peak}=\frac{1}{{\left({\rho }_{o2}{\rho }_{c}{\rho }_{+}\right)}^\frac{1}{3}}$	${\tau }_{peak}=\frac{1}{{\left({\rho }_{o2}{\rho }_{+}\right)}^\frac{1}{2}}\sqrt{\frac{{\rho }_{e}}{{\rho }_{c}}}$
Persistence	${\tau }_{decay}\simeq \frac{1}{{\rho }_{c}+{\rho }_{d}}$	${\tau }_{decay}\simeq \frac{1}{{\left({\rho }_{o2}{\rho }_{+}\right)}^\frac{1}{2}}\sqrt{\frac{{\rho }_{e}}{{\rho }_{c}}}$
Productivity	$p=\frac{{\rho }_{c}}{{\rho }_{e}}$	$p=\frac{{\rho }_{c}}{{\rho }_{e}}$
Abundance	$\frac{{c}_{peak}}{m\left(0\right)}=\frac{{\rho }_{o2}}{{\rho }_{e}}$	$\frac{{c}_{peak}}{m\left(0\right)}=\frac{{\rho }_{o2}}{{\rho }_{e}}$
***Primary nucleation-dominated***		
Peak time	${\tau }_{peak}\simeq \frac{1}{{\left({\rho }_{o1}{\rho }_{+}\right)}^\frac{1}{2}}\sqrt{\frac{{\rho }_{e}}{{\rho }_{c}}}$, or $\frac{1}{\sqrt{{\rho }_{+}{k}_{-}}}$ (if fragmentation dominated)	
Persistence	${\tau }_{decay}\simeq \frac{1}{{\rho }_{c}+{\rho }_{d}}$	${\tau }_{decay}\simeq \frac{1}{{\left({\rho }_{o1}{\rho }_{+}\right)}^\frac{1}{2}}\sqrt{\frac{{\rho }_{e}}{{\rho }_{c}}}$
Productivity	$p=\frac{{\rho }_{c}}{{\rho }_{e}}$	$p=\frac{{\rho }_{c}}{{\rho }_{e}}$
Abundance	$\frac{{c}_{peak}}{m\left(0\right)}=\frac{{\rho }_{o1}}{{\rho }_{e}}$	$\frac{{c}_{peak}}{m\left(0\right)}=\frac{{\rho }_{o1}}{{\rho }_{e}}$

### Amyloid aggregation within protein condensates

The process by which proteins form a liquid-like condensed phase is also becoming increasingly recognized as relevant in both physiology [132, 215–217] and pathology [218, 219]. This phenomenon, which is known as protein phase separation (PPS), may take place when protein–protein interactions become more favorable than protein-solvent interactions [217]. Driving forces for this process include electrostatic interactions (cation–anion, dipole–dipole cation-π, sp2-π, π-π), polar interactions mediated by hydrogen bonds, and hydrophobic interactions, amongst others [220–223]. The environmental conditions influence greatly protein phase separation, where factors like protein concentration, RNA interaction partners, co-solutes, temperature, pH, salt type and concentration, and crowding agents can drastically change the way in which proteins interact with each other. Although most proteins appear capable of protein phase separation [132], we are only beginning to understand the sequence-based determinants of the propensities of different proteins to do so [223–228].

The presence of liquid-like condensates opens the possibility of an alternative pathway to amyloid aggregation. This “condensation pathway” is distinct from the direct formation of amyloid aggregates from the native state occurring through oligomeric species, which is known as the “deposition pathway”. Formation of solid deposits either directly through the deposition pathway or from liquid droplets through the condensation pathway have been observed even within the same cell and protein system undergoing self-assembly [229–232].

The role of oligomers is not yet clear, however, when amyloid aggregation takes place within condensates. TDP-43 oligomers were found to form in both the deposition and condensation pathways, and to form before solid aggregates emerge within a condensed gel-like phase [233]. It has also been reported that ⍺-synuclein oligomers formed immediately following phase separation of the monomeric protein into a hydrogel, where monomers, oligomers and fibrils co-exist and where the hydrogels entrap, rather than release, oligomeric and fibrillar ⍺-synuclein in a highly cytotoxic state [234]. Similarly, it was found for tau that liquid–liquid phase separation precedes gel formation and then aggregation in vitro [235]*,* and induces a pathogenic conformation and oligomerization [235, 236]. Another RNA-binding protein, TIA1, further potentiates tau phase separation, facilitating the oligomerization and subsequent cytotoxicity of the microtubule-associated protein [237]. The investigation of a coarse-grained peptide also found the formation of both metastable and stable oligomers in a dense phase [238].

## Oligomer detection methods

Although oligomers are aberrant assemblies that can interact with a wide range of cellular components [1, 100], their concentration remains low during the amyloid aggregation process. For example, at the half-point of an aggregation reaction in vitro where half the monomers have converted into fibrils, the concentration of oligomers can be two or three orders of magnitude lower than the monomer concentration [21]. It is therefore often necessary to isolate oligomers at higher concentrations or stabilize them to facilitate their investigation in vitro [161, 239–241]. It is important to note that on-pathway oligomers are the focus of the kinetic models described above, whereas stabilization methods typically redirect the aggregation reaction towards the formation of off-pathway oligomers at experimentally amenable concentrations.

Many techniques are available for the detection of oligomers [115]. Immunoassays are commonly used and are based on conformation- or sequence-specific antibodies that capture and trap oligomers in solution, ideally without appreciably detecting monomers or fibrils [118, 242]. Examples include the A11 polyclonal antibody to detect prefibrillar spherical oligomers by Aβ and other systems [118], the OC polyclonal antibody against Aβ fibrillar oligomers [120], the polyclonal M94 antiserum against Aβ ADDLs [243], ASyO2 to bind 600 kDa ⍺-syn oligomers [244], mAB-O that binds 25–150 kDa Aβ_42_ oligomers [244], 71A1 to bind 670 + kDa Aβ oligomers [245], and another that binds Aβ oligomers markedly more so than its monomers or fibrils [246]. Importantly, antibodies of this type have been used to detect oligomers in AD brains that were absent in aged-matched healthy individuals [118, 120, 243].

A variety of antibody-based assays are used for detecting oligomers in various samples [115, 123, 247]. However, generating antibodies with high specificity for oligomers remains challenging, as many antibodies initially reported to be oligomer-specific often do not differentiate well between oligomers and fibrils [248]. Despite this, developments have been made with biosensors [115], which allow for a label-free capture and detection of oligomers through antibodies. Biolayer interferometry (BLI) uses oligomer-specific antibodies attached to glass fibre tips for their functionalization [249]. Using surface plasmon resonance (SPR), interactions between immobilized conformation-specific antibodies and oligomers are observable through changes in reflected light. SPR can be end-coupled to mass-spectrometry for further characterization (i.e., mass, stoichiometry, topology, charge, etc.) and quantification of oligomers [250–254]. Other oligomer detection techniques include dye-derived fluorescence spectroscopy and microscopy, electron microscopy, atomic force microscopy (AFM), DLS, filter-trap assays, radiolabeling, mass photometry, and numerous others [115].

### Characterization methods of oligomer structure

The physicochemical properties of oligomers are important mediators of their cytotoxicity. A variety of experimental techniques have been developed to monitor these properties, some of which are discussed here. Hydrophobicity is readily quantified in certain experimental settings using 8-anilinonaphthalene-1-sulfonate (ANS) florescence (or its derivative bis-ANS), as its intensity increases and its wavelength of maximum fluorescence undergoes a blue shift upon binding to hydrophobic regions that are solvent exposed in a protein [255]. Oligomers induce these changes more than fibrils, whereas monomers typically exhibit minimal changes in ANS fluorescence [241].

Oligomer size can be quantified through a wide array of methods with varying levels of sensitivity, including microfluidic diffusional sizing [246, 256], single-molecule TIRF and dSTORM super-resolution imaging [246], static and dynamic light scattering (SLS and DLS, respectively) [257–259], size-exclusion chromatography [91], several types of polyacrylamide gel electrophoresis [260], photo-induced cross-linking of unmodified protein (PICUP) [261], AFM [262], and cryo-EM [161].

The secondary structure of oligomers has also been widely investigated, for example using circular dichroism (CD) and Fourier transform infrared (FTIR) spectroscopy [241]. Based on these studies, our current understanding is that secondary structure is not clearly linked to oligomer toxicity [263], unlike size and hydrophobicity. Analogous to the case for ANS binding, CD and FTIR spectra differ significantly for oligomers in comparison to monomers or fibrils. While ANS binding tends to be higher for oligomers, the secondary structure of oligomers assessed by CD or FTIR tends to be intermediate between monomeric and fibrillar preparations.

Site-specific structural information has been gained using a variety of experimental approaches. Protein engineering to substitute a given residue to cysteine has been used to label the same residue with a probe that is either fluorescent or paramagnetic and reporting on a specific structural type of information, such as solvent exposure, degree of packing, and distance from another residue via fluorescence resonance energy transfer (FRET), using either fluorescence or electron paramagnetic resonance (EPR) [240, 264–266]. Proline scanning mutagenesis has also been used to scan the involvement of any residue in oligomer structure [267]. Various applications of solution and solid-state nuclear magnetic resonance (NMR) spectroscopy have been utilized to map out residues with β-sheet structure within the oligomers [33, 154, 165, 266]. Further advancement awaits technological progress in applications of cryo-EM, which is not as advanced in providing oligomer structures as it is on fibril structures.

## Oligomers in protein misfolding diseases

Despite a quest that has already lasted over two decades, it has been difficult to obtain direct evidence that misfolded protein oligomers are cytotoxic species in protein misfolding diseases. While there is a wealth of information about the cytotoxicity of oligomers in vitro on cultured cells, primary neurons and brain slices using a variety of peptide and protein systems and a variety of biological observables, it has proven much more challenging to establish direct links between oligomer formation, cellular dysfunction and disease phenotype in vivo [104]. Perhaps one of the strongest elements of support for the oligomer toxicity hypothesis comes from recent clinical trials on AD patients, and consequent Food and Drug Administration (FDA) accelerated and then traditional approval to treat AD, of lecanemab, an antibody raised against high molecular weight Aβ oligomers, also known as soluble protofibrils, which has shown that targeting these species slows down cognitive decline in AD human cases [268], and reduces brain and CSF Aβ oligomers in a mouse model [269]**.** The mechanism of action of lecanemab is consistent with that of aducanumab [270, 271], another, but more controversial, antibody approved by the FDA for AD treatment, which has been shown to target mature forms of Aβ and reduce oligomer formation by inhibiting secondary nucleation in vitro [272]. In particular, lecanemab, aducanumab, and gantenerumab have been characterized to bind differentially to various Aβ species, where lecanemab demonstrated a 10-fold stronger binding affinity for protofibrils over fibrils, and aducanumab and gantenerumab showed prefferential binding to fibrils instead of protofibrils [273]. It was also shown that all three antibodies had a low affinity for monomers, but lecanemab and aducanumab showed very weak monomer binding [273].

Well before these achievements, the hypothesis that small oligomers, as opposed to fibrils, are the main pathogenic species in AD had been supported by many lines of circumstantial evidence [1, 100, 102, 103]. These include: (1) the higher cytotoxicity and synaptotoxicity of oligomers to cultured cells, primary neurons, and iPSC-derived human neurons [50, 100, 274], (2) impairment of social memory, reduced brain volume, increased caspase-3-positive cells, microglial and pro-inflammatory responses, following the injection of human Aβ in rat brains [275], (3) evidence that severity of AD and synaptic loss does not correlate with the extent of amyloid plaque formation, but with the biochemically detected amount of soluble Aβ (including soluble oligomers) [276], (4) observations that in some transgenic APP mouse models, biochemical and electrophysiological evidence of synaptic alteration and cognitive impairment precedes amyloid plaque formation, but occurs after Aβ levels start to rise steadily [277], and (5) the E693G mutation associated with familial early AD promotes protofibril rather than fibril formation [278].

One of the reasons for the lack of direct evidence of a causal role of oligomers in disease is the difficulties in isolating oligomers from post-mortem brain tissue due to their small size, low stabilities, low concentrations, transient nature, and extensive structural heterogeneity. Structures for toxic oligomers have been determined from in vitro preparations and were cylindrical in shape, including for example ⍺B-crystallin [279] and ⍺-synuclein [161]. While there are over 250 amyloid fibril structures resolved by ssNMR or cryo-EM in the Amyloid Atlas [137], a key challenge remains analyzing the structural motifs of the oligomers present in human pathology. It is clear that different fibril polymorphs are associated with different diseases and phenotypes, but it remains to be determined if this phenomenon holds true also for oligomeric aggregates. Moreover, supernatants of high-speed ultracentrifugation preparations from aqueous AD brain extracts have been recently reported to contain fibrils with the same structure as those from plaques, and these fibrils bound lecanemab resulting in their diminished synaptotoxicity [280].

Nevertheless, conformation-sensitive antibodies have allowed the detection of well-defined oligomers in AD and PD patients relative to aged-matched controls [43–45, 121, 243, 281–286] and increased levels of oligomers have been observed in the CSF of AD [284, 287] and PD cases [45, 288, 289], although these observations do not stand per se as a conclusive proof that oligomers are the causative agents of protein misfolding diseases. Recently, soluble protein aggregates were isolated from eight brain regions for AD patients at Braak stage III [290]. Soluble aggregates approximately 2 nm in diameter and less than 100 nm in length from all these regions were neuroinflammatory and permeabilized liposomes to varying extents, suggesting that this early stage of disease is characterized by a global pathology occurring to differing extents in various regions but simultaneously in the entire brain. Another study using gentle extraction methods rather than conventional brain tissue homogenization found that only a critical minority of Aβ consisted of diffusible oligomers, which were responsible for inducing toxicity [291].

Comparing the CSF of individuals with AD, mild cognitive impairment (MCI), and healthy controls, MCI cases demonstrated a greater extent of small aggregates that could induce membrane permeabilization, while AD individuals exhibited larger aggregates that robustly triggered pro-inflammatory responses in glial cells [292]. These results in part suggest that the number and size distributions of aggregates evolves over time during the progression of AD, the latter of which could be quantified in CSF samples [292].

### Oligomer toxicity in animal models

Oligomers have been investigated in transgenic animal models, including Aβ oligomers [293–295] and ⍺-synuclein oligomers [296, 297]. Many studies have linked these small aggregates in particular to the onset and development of cellular dysfunction and neurodegeneration [31, 43, 100, 298, 299]. APP transgenic mice with the E693 delta mutation exhibit extensive Aβ oligomerization without fibril formation alongside impairments to synaptic plasticity and memory, abnormal tau phosphorylation, microglial and astrocyte activation, and neuronal loss at varying time points from 8–24 months [294]. Injection of oligomers from various sources in mice or rat brains resulted in severe impairments. For example, toxic misfolded HypF-N oligomers injected into rodent brains caused loss of cholinergic neurons, spatial memory impairments, synaptic colocalization in primary neurons, and attenuated long-term potentiation (LTP) in hippocampal brain slices [274, 300]. Tau oligomers injected into the wild-type mouse brain also triggered synaptic and mitochondrial dysfunction [18]. Natural Aβ oligomers formed within specific intracellular vesicles and subsequently secreted extracellularly were also found to be effective at inhibiting LTP in rats in vivo upon cerebral microinjection [301]. Oligomeric Aβ also markedly potentiated intracellular Ca^2+^ ion influx upon the exogenous treatment of healthy mice brains with soluble Aβ oligomers [302]**.** In addition, Aβ oligomers triggered tau pathology, caused synaptic loss and axon transport dysfunction, insulin resistance, cholinergic impairment, choline acetyltransferase inhibition, neuroinflammation, and epigenetic changes [284, 303]. Injection of toxic α-synuclein type B* oligomers into the mouse striatum induced a small but significant loss of dopaminergic neurons in the *substantia nigra pars compacta*, although a higher effect was found when injecting small short fibrils of the same protein as a result of the ability of fibrils to spread and amplify ⍺-synuclein aggregation [304]**.**

### Oligomer interactions with cellular targets

Misfolded protein oligomers have been shown to bind generically to biological membranes resulting in a toxic gain of function, to specific membrane receptors resulting in a loss of native function, and to cytosolic proteins and nucleic acids, wherein these mechanisms can occur simultaneously [1]. It is unlikely that a single molecular interaction, mechanism of action or cellular cascade is responsible for causing pathology in protein misfolding diseases [1]. Rather, the toxicity of protein aggregates, including misfolded protein oligomers, is likely a consequence of their misfolded structure and extensive heterogeneity, which enables them to induce a wide range of dysfunctional cellular interactions in a litany of cellular compartments, including lipid bilayers, discrete receptors, soluble proteins, RNAs, and metabolites and culminating in cell death [1]. In the last sections of review, we consider the role that oligomeric species play in the events associated with amyloid-associated cytotoxicity, with a focus on how oligomers interact with cells, the cellular consequences of these interactions, and a subsequent discussion of therapeutic efforts aimed at these approaches.

**Fig. 6:**
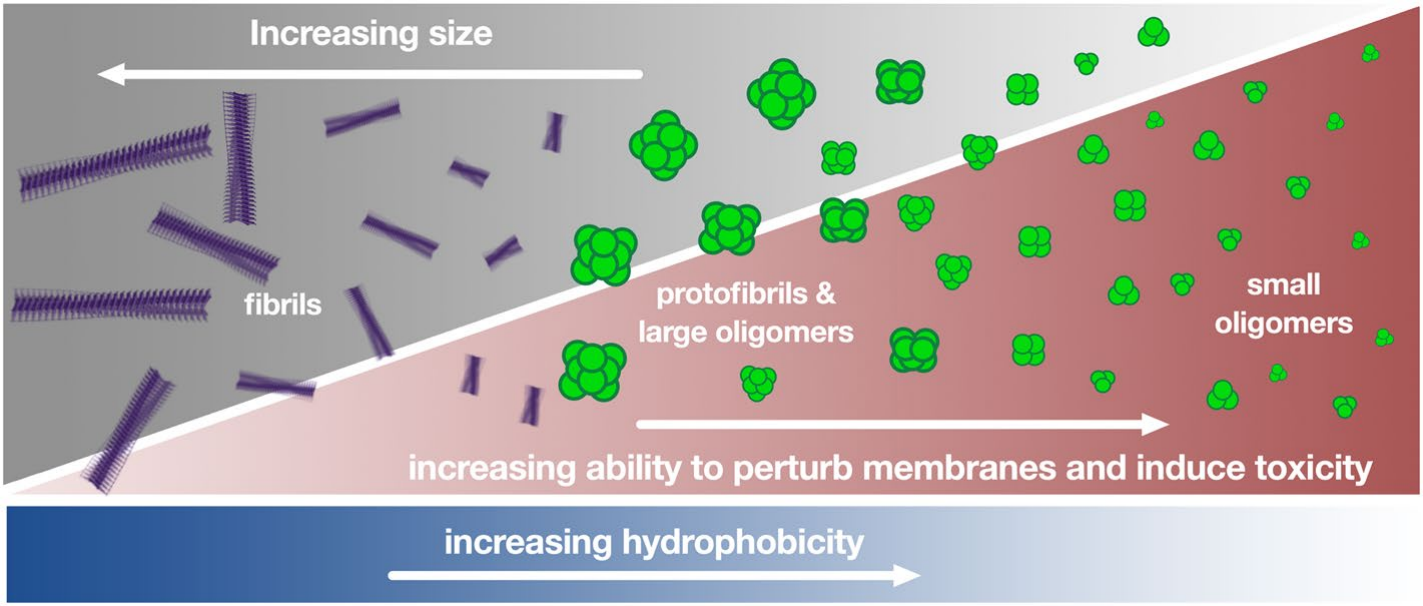
Physicochemical parameters that influence the toxicity of protein misfolded oligomers. The toxicity typically scales with increasing hydrophobicity and decreasing size. Created with biorender.com

Membrane disruption caused by protein misfolded oligomers leads to neurotoxicity characterized by calcium imbalance, mitochondrial dysfunction, and intracellular reactive oxygens species (ROS) production [240, 305, 306]. With respect to how oligomers induce membrane perturbation, which can also be accomplished by fibrils albeit often to a lower extent [106, 108, 307], a clear relationship exists between size, hydrophobicity and toxicity of misfolded protein oligomers, where oligomers that are small and have a greater extent of hydrophobic amino acids being solved exposed are the most cytotoxic (Fig. 6). Small oligomers have greater diffusional mobility and therefore reach the cell membrane more frequently [308, 309], while oligomers with enhanced hydrophobicity are able to embed and readily insert into the interior of lipid bilayers [33, 160, 240, 264, 310, 311], therein perturbing the membrane and inducing toxicity. High molecular weight oligomers isolated from AD brains were found to be only mildly neurotoxic, whereas their dissociation into lower molecular weight oligomers in mildly alkaline buffer markedly increased their toxicities [312].

Molecular chaperones have been shown to alleviate oligomer toxicity by increasing their size [308]. A size-toxicity relationship has been established for Aβ aggregates, where low molecular weight oligomers are markedly more cytotoxic than larger oligomers and fibrillar aggregates display the least toxicity [100]. By isolating Aβ_42_ soluble aggregates of different sizes using gradient ultracentrifugation, smaller soluble aggregates were found to more overtly induce membrane permeabilization, suggesting an inverse relationship between size and toxicity, while larger soluble aggregates more potently induced an inflammatory response in microglia cells [313]. Recently, small soluble aggregates of ⍺-synuclein less than 100-200 nm were identified as the toxic species in PD through the comparison of in vitro oligomers to soluble aggregates in post-mortem PD brains [32].

The importance of the hydrophobicity-toxicity connection is exemplified from the observation that oligomers of similar size and dissimilar hydrophobicity can be experimentally stabilized, where only the oligomers of greater solvent exposed hydrophobicity are capable of inducing significant levels of cellular toxicity and dysfunction. This phenomenon has been observed for pairs of toxic and nontoxic oligomers [116] for HypF-N [240, 264], sup35NM [311], ⍺-synuclein [33, 160], and Aβ [310].

In addition to the properties of oligomers that can mitigate their binding to cell membranes, the composition of the cell membranes themselves plays an important role in oligomer binding and in the induction of toxicity [314]. In fact, cell membranes enriched in the monosialotetrahexosylganglioside GM1, which is abundant in lipid rafts alongside cholesterol and sphingomyelin, exhibit heightened Aβ_42_ and HypF-N oligomer binding [306]. A key finding of that study is that the quantified toxicity was directly proportional to the extent of oligomer binding [306].

Beside interacting with biological membranes, misfolded protein oligomers have been reported to interact with, or modulate the activity of, a variety of cellular components and receptors, including the cellular form of the prion protein (PrP^C^) [315], alpha7 nicotinic acetylcholine receptor (⍺7-nAChR) [316, 317], low-density lipoprotein receptor-related protein-1 (LRP1) [315, 318], and many others. In particular, Aβ oligomers have been described to interact with over 20 types of receptors [319] and also extracellular and intracellular synaptic proteins, including Na/K-ATPase, synGap, and Shank3 [320]. Calcium dyshomeostasis has been associated with AD via overactivation of glutamatergic receptors, and Aβ oligomers have been found to activate to a small extent AMPA receptors and to a large extent NMDA receptors resulting in the rapid influx of calcium ions into the cytoplasm [321]. The misfolded oligomers interact indirectly with these receptors, and the activation was caused by oligomer-induced changes in membrane tension that were sensed by mechanosensitive NMDA and AMPA receptors [321]. Specific receptors for Aβ oligomers were shown to recognize features of both toxic oligomers and fibril ends. In particular, PrP^C^, Fcγ receptor IIb (FcγRIIb), and leukocyte immunoglobulin-like receptor B2 (LilrB2) were characterized to bind the ends of fibrils, neurotoxic oligomers, and protofibrils, therein inhibiting fibril growth [322].

**Fig. 7:**
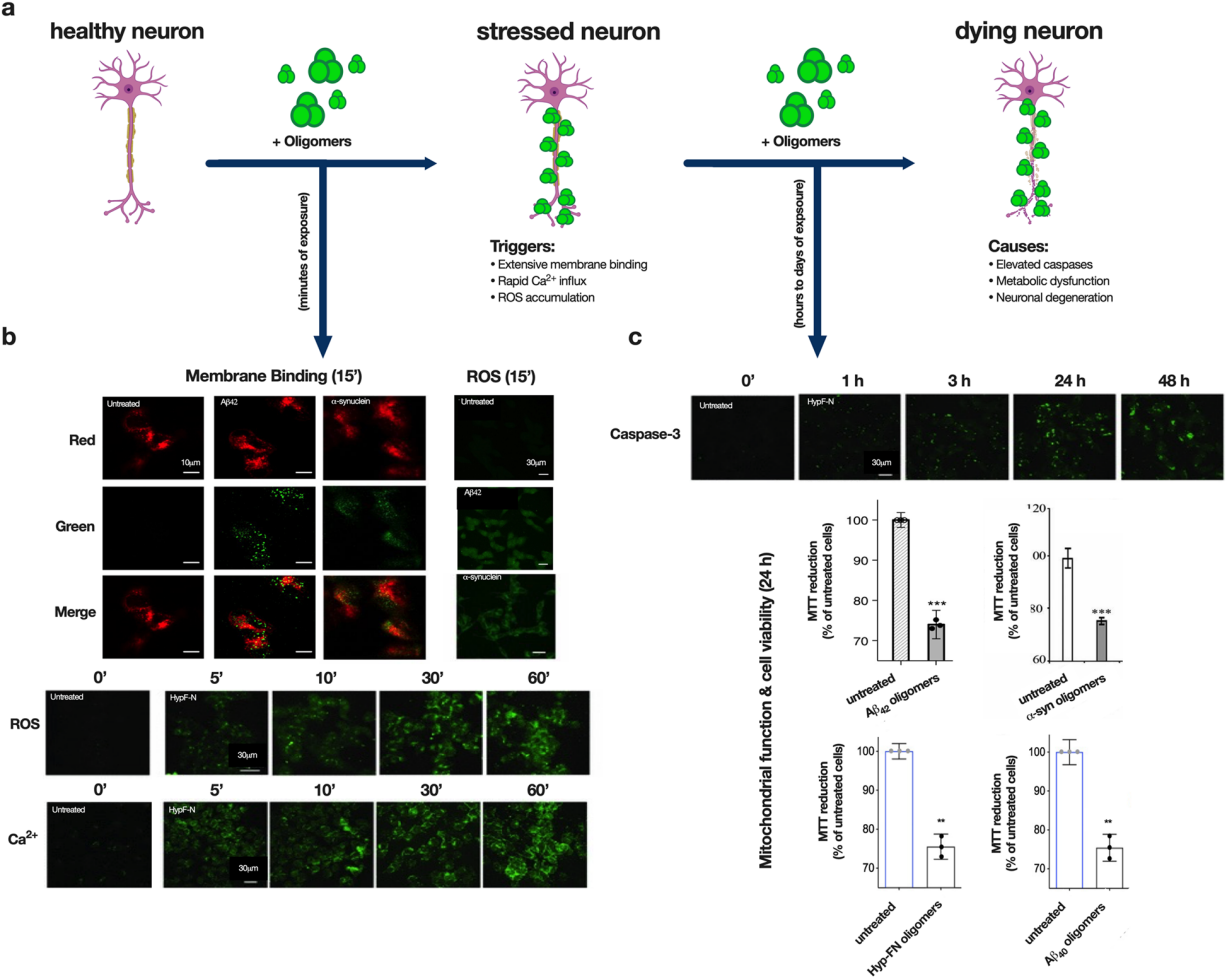
Oligomers induce cytotoxic effects that can be monitored over time. **a** In healthy cells, over short durations (minutes up to one hour), toxic oligomers exhibit extensive membrane binding, induce rapid influx of Ca^2+^ ions, and then reactive oxygen species (ROS) accumulation [321]. Longer incubations (hours to days) induce elevated caspase-3 levels, metabolic dysfunction, and ultimately death of the cell [331]. Created with biorender.com. **b** Examples of observable impacts of Aβ_42_ and ⍺-synuclein oligomer treatment for short durations to SH-SY5Y human neuroblastoma cells [257, 332]. Membrane binding: oligomers (green chancel) and membranes (red channel) [257]. ROS production (green). Intracellular calcium ions (green). **c** Examples of observable impacts of HypF-N oligomer treatment for longer durations (hours to days) to SH-SY5Y cells, including caspase-3 production (green) [300] and metabolic defects as assessed using the MTT assay for Aβ_40_, Aβ_42_, ⍺-synuclein, and HypF-N oligomers [257, 258, 332]. Panels were adapted from Limbocker et. al [257], Zampagni et.al [300], Perni et. al [332], and Limbocker et. al [258]

While Aβ oligomers can bind membrane bilayers and proteins and trigger aberrant intracellular cascades, evidence also suggests that Aβ oligomer formation in the cell, or internalization into the cell, is also important. Aβ aggregation induced by cell uptake contributes to cell death and culminates in the release of amyloid aggregates outside the cell [323]. Aβ can enter the cell by pore formation, endocytosis, and via specific receptors [324–326]. Moreover, oligomers can initiate aberrant protein–protein interactions. The hyperphosphorylation of tau and its aggregation, for example, can potentiate Aβ oligomer-induced dysfunction in AMPA receptor signaling [327]. Aβ oligomers have also been shown to promote the internalization of fibrillar tau seeds resulting in increased intracellular tau aggregation [328]. Different conformations of neurodegeneration-linked proteins can also impact one another. For example, ⍺-synuclein monomers inhibit Aβ_42_ secondary nucleation, whereas fibrillar ⍺-synuclein stimulates Aβ_42_ heterogeneous nucleation [329]. Pathological ⍺-synuclein accumulation was recently shown to disrupt the decapping module of P-bodies, therein disrupting mRNA stability in iPSC-derived neuronal models of PD [330]. Figure 7 summarizes a subset of the deleterious interactions and effects that results from the interaction between cells and misfolded protein oligomers.

### Cellular consequences of oligomer interactions

**Fig. 8:**
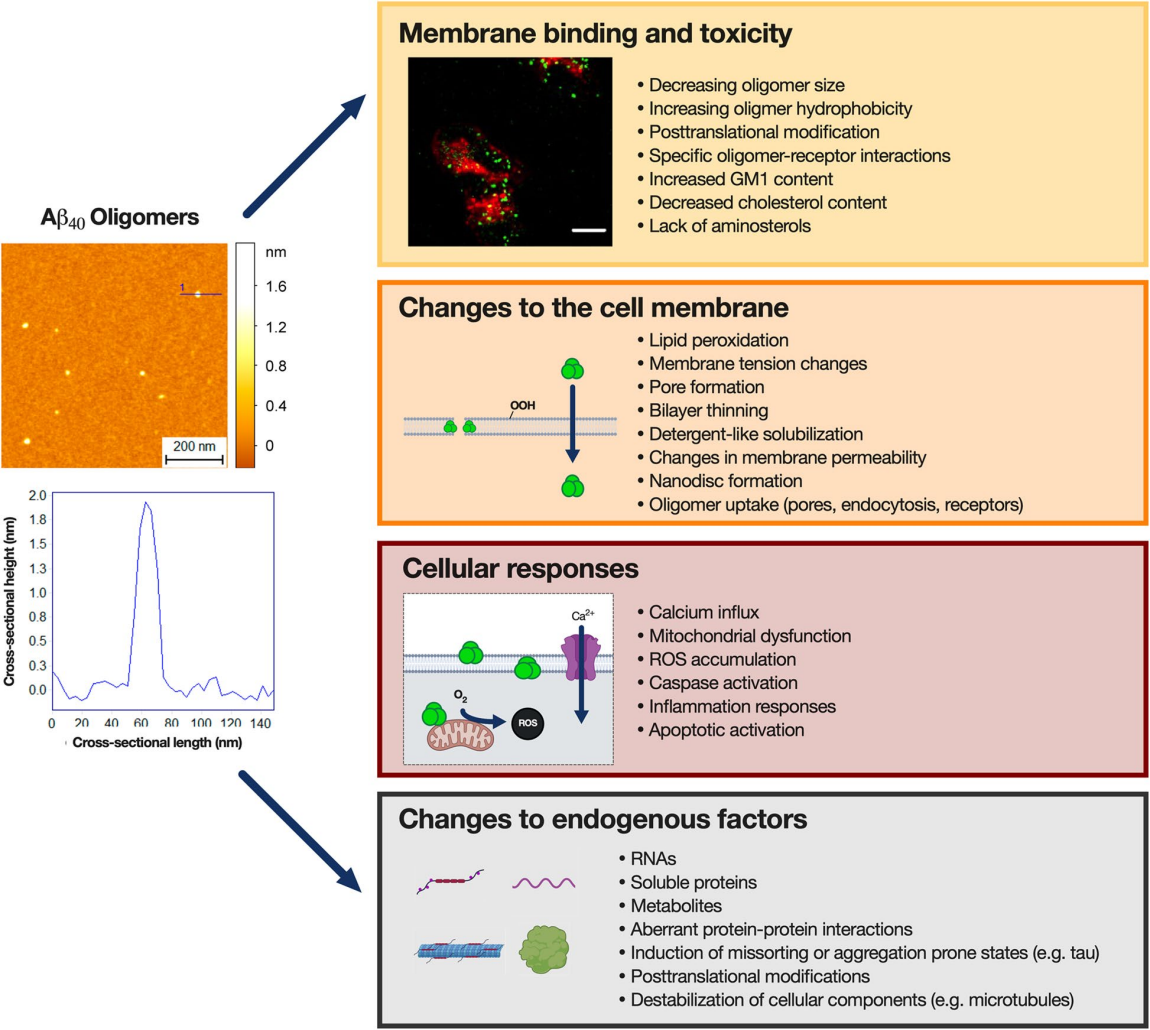
Consequences of the exposure of cells to misfolded protein oligomers. AFM cross-sectional profile of Aβ40 oligomers stabilized by Zn^2+^. Misfolded protein oligomers of this type typically are 2–6 nm in height, and they induce membrane binding and toxicity accompanied with deleterious changes to the properties of cell membranes, cellular responses, and changes to endogenous factors. The AFM map and cross-sectional profile were adapted from Limbocker et. al [337]. The membrane binding panel was adapted from Limbocker et. al [257]. Created with biorender.com

Toxic misfolded protein oligomers demonstrate a preferential ability to penetrate the lipid bilayers of cell membranes (Fig. 8) [116]. These interactions trigger numerous events, including calcium influx into the cell, intracellular production of ROS, lipid peroxidation, cell membrane leaking and the escape of intracellular molecules, caspase-3 activation, and mitochondrial damage [116, 240, 300, 305, 333, 334]. Among the earliest events that take place after misfolded protein oligomer binding to the cell are the influx of calcium ions from the extracellular medium that is mediated by NMDA receptors. Oligomeric Aβ, for example, is known to markedly potentiate intracellular Ca^2+^ influx in cell culture [306, 321, 335, 336], which then triggers ROS accumulation and following events [335]. Changes in Ca^2+^ levels precede synaptic damage in vivo [302].

Another hallmark of neurodegenerative diseases arising from misfolded proteins is the presence of elevated levels of ROS [338, 339]. Misfolded protein oligomers are able to induce extensive ROS generation leading to a cascade of intracellular consequences, such as ROS-mediated activation of apoptosis signal-regulating kinase 1 (ASK1) associated with the toxicity of Aβ in AD [340]. High levels of ROS can also stimulate lipid peroxidation of cell membranes, which in turn can impact protein aggregation and lead to the loss of organelle function [341]. In cardiomyopathy, the deposition of transthyretin (TTR) increased the production of ROS, correlating with left ventricular systolic dysfunction, though likely indirectly from endoplasmic reticulum stress and calcium dyshomeostasis by non-fibrillar TTR species [89, 342–344].

Mitochondrial dysfunction is also associated with the presence of misfolded protein oligomers [345]. Oligomers or protofibrils have been shown to perturb mitochondrial membranes and induce significantly the influx of Ca^2+^ [240, 321], interrupt normal metabolic processes during oxidative phosphorylation [345], and trigger apoptotic pathways [346, 347]. For example, IAPP oligomers associated with type II diabetes are toxic by increasing Ca^2+^ influx [336] and disrupting the mitochondrial membrane in pancreatic β-cells [78].

In addition, Aβ oligomers can also interact with proteins or lipids at synapses and inhibit LTP, which is a correlate of synaptic plasticity [291]. They can also induce an inflammatory response in microglial cells upregulating, among other factors, the major histocompatibility complex class II, inducible nitric oxide synthase, and CD40 in the hippocampus of AD transgenic mice [348] and release pro-inflammatory cytokines [349]. Several key dysfunctional responses caused by oligomers to cell membranes, whole cells and endogenous factors are summarized in Fig. 8.

## Therapeutic approaches targeting misfolded protein oligomers

Numerous strategies have been studied in vitro to attenuate the toxicity of misfolded protein oligomers, including reducing their concentration and lifetime, increasing their size, neutralizing their hydrophobic surface, targeting their toxic interactions with molecular targets, such as specific receptors or cell membranes, or enhancing their clearance [101, 309, 350]. As covered in the previous sections, misfolded oligomers can induce cytotoxicity in many ways, which is in large part a result of their intrinsic heterogeneity and ability to interact with a wide variety of cellular components. While it is critical to understand the diverse means by which these oligomers can damage many parts of the cell, it is unlikely that blocking specific oligomer-target interactions will be sufficient to arrest the toxicity of amyloid pathologies. The clinical relevance of many of the strategies discussed here will become clearer over the next few decades by building upon the momentum of the recent successes obtained with monoclonal antibodies for the treatment of AD.

### Reduction of oligomer formation

The protein homeostasis system is capable of inhibiting specific microscopic steps of an aggregation process using molecular chaperones, such as ⍺B-crystallin that inhibits ⍺-synuclein and Aβ elongation or Hsp70 that can inhibit tau primary nucleation as well as sequester oligomeric and mature tau into inert and seeding-incompetent species [351–354]. This strategy has been extensively investigated as a therapeutic approach for protein misfolding diseases with small molecules [355, 356]. A challenge, however, is that there is no direct relationship between the reduction in the number of amyloid aggregates and the reduction in the number of oligomers [114]. For example, a fibril elongation inhibitor delays the aggregation process, but contributes to the accumulation of oligomers [114]. It has been estimated that the inhibition of fibril elongation by one order of magnitude can generate a five-fold increase in the concentration of oligomers [212].

The inhibition of primary nucleation may offer opportunities for therapy, as it delays formation of oligomeric species (Fig. 5). In fact, several candidates have been identified for primary nucleation inhibition [332, 357–362]. Of great promise, targeting secondary nucleation would be particularly beneficial therapeutically as it causes the autocatalytic proliferation of aggregates and is primarily responsible for oligomer production (Fig. 5) [21, 114, 363]. This approach has been realized by several molecular agents, including a group of small molecules [364] and antibodies [365] that target secondary nucleation in Aβ_42_ aggregation to differing extents. A similar approach was utilized to evaluate a library of flavones against ⍺-synuclein aggregation, with focus on drugs that most inhibit oligomer formation [179]. Moreover, specific aminosterols can inhibit fibril amplification secondary processes in ⍺-synuclein aggregation [362, 366]. Molecular chaperones can also target secondary nucleation in Aβ_42_ aggregation [354, 360]. Structure-kinetic activity relationship (SKAR) rules have also been leveraged to convert an inactive rhodamine molecule into a derivative that could inhibit secondary nucleation in Aβ_42_ aggregation [113].

**Fig. 9:**
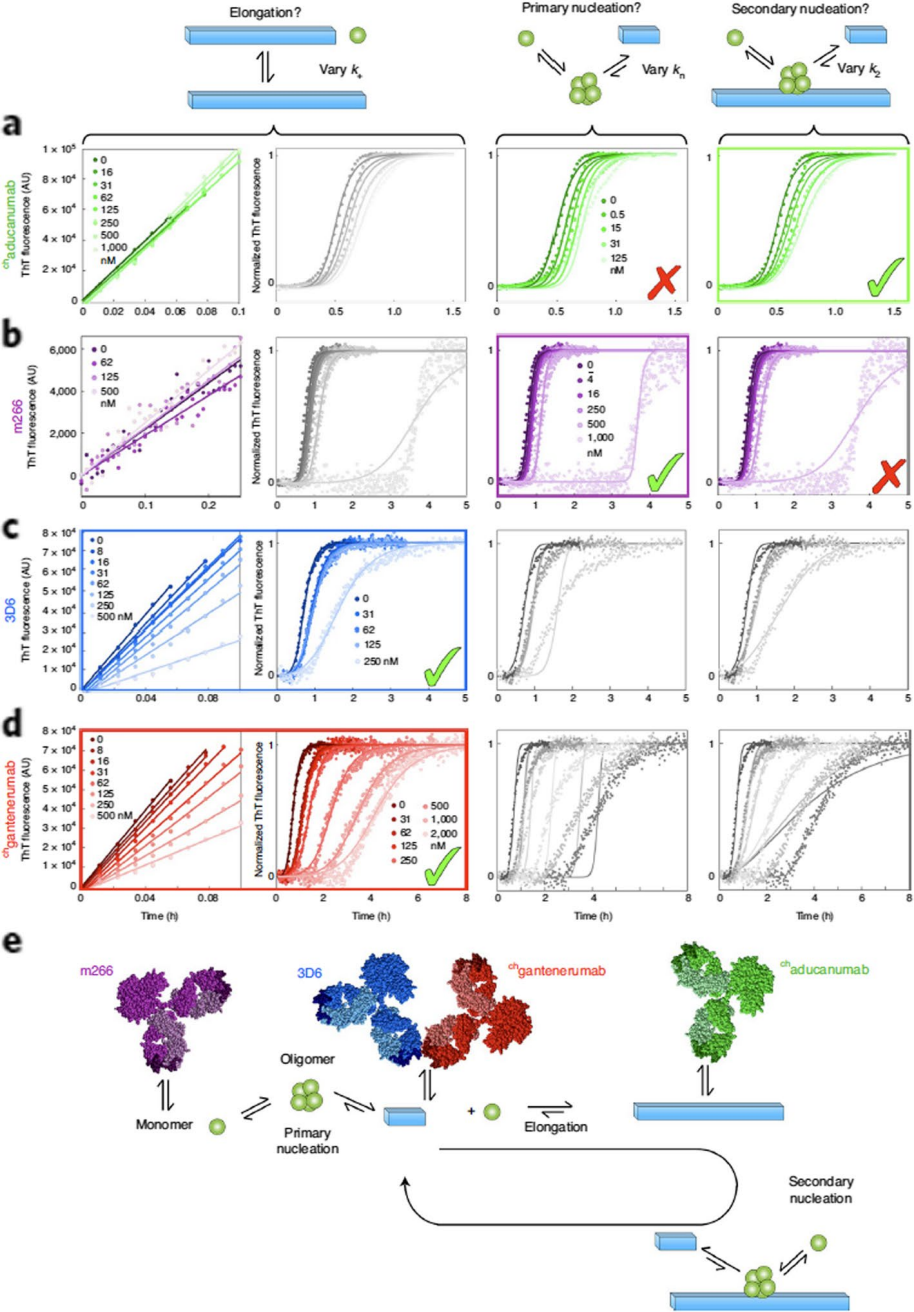
Clinical-stage antibodies against AD target different microscopic steps in Aβ_42_ aggregation. From Linse et. al [272]

A kinetic analysis of four anti-Aβ antibodies in different stages of clinical trials at the time of that publication found that aducanumab (granted accelerated approval by the FDA [367]) targets secondary nucleation in the Aβ_42_ aggregation process and therefore the reactive flux of oligomers, whereas bapineuzumab, solanezumab, and gantenerumab impacted other microscopic processes including elongation or primary nucleation (Fig. 9) [272]. Of interest, the monoclonal antibody ACU193 is suggested to be an Aβ oligomer-selective immunotherapeutic and is in clinical trials [368].

### Neutralization of oligomers through binding

Compounds that bind directly misfolded oligomers have also been identified and their mechanism has been investigated. While binding oligomers can have the effect of stabilizing them, it can also neutralize their hydrophobic surfaces, inhibit their action as nuclei, or remodel the aggregation pathway towards the formation of less toxic species. Phage display was used to identify a soluble inhibitor specific to Aβ_42_ oligomers and able to inhibit their ability to act as nuclei [369]. Similarly, a rational design strategy was used to obtain oligomer-specific antibodies that undermine secondary nucleation [246]. It is also possible to drive off-pathway aggregation processes with small molecules, as observed for the polyphenol (-)-epigallocatechin gallate (EGCG) that can form oligomers of ⍺-synuclein and Aβ that are non-toxic [370] or resveratrol that can remodel Aβ_42_ into nontoxic, high molecular weight species [371].

### Stabilization of the native state

Another approach to prevent aggregation is the stabilization of the native state of proteins. This approach has been implemented in the case of protein misfolding diseases caused by amyloid aggregation of transthyretin, through the small molecule tafamidis [372, 373], which was approved by the European Medicines Agency (EMA) in 2011 for the treatment of stage I ATTRv-polyneuropathy, after successful completion of a phase III clinical trial [374]. Then, in 2019 and 2020, the drug was approved by the FDA and EMA, respectively, for the treatment of ATTRv- and ATTRwt-cardiomyopathy at stages I and II, after a successful phase III clinical trial was noted [375]. Tafamidis was the first approved drug to slow down the progression of an amyloid disease. A similar approach is also being explored for light chain amyloidosis (AL) by using small molecules that bind at the native monomer–monomer interface of native dimeric immunoglobulin light chains [376]. Native state stabilization has been shown to be effective more generally for three other protein misfolding diseases that are not associated with amyloid formation, through the use of drugs is approved in all three cases by both FDA and EMA [377]. This approach, however, is challenging for disordered proteins, such as Aβ, tau and ⍺–synuclein, since these proteins do not exhibit stable binding pockets for small molecules [378, 379]. Binding mechanisms in which the contribution to the free energy of binding comes from entropy have been explored [380]. The small molecule 10074-G5 was recently found to increase the conformational entropy of monomeric Aβ alongside decreasing its hydrophobic exposure, resulting in the stabilization of the monomeric state and the prevention of aggregation [378].

### Oligomer clearance

Other promising therapies are being explored to potentiate oligomer degradation through a variety of mechanisms, including activating the unfolded protein response, stimulating autophagy, aiding extracellular clearance, rebalancing the proteostasis network (PN) by targeting specific heat shock factors, and exploiting molecular chaperones working as disaggregases [381]. However, approaches based on the exploitation of the PN are beyond the scope of the present review. Aβ oligomer clearance by passive immunization is of course a very promising strategy, as shown by the recent accelerated and then regular approval by the FDA of lecanemab, designed to target mainly soluble Aβ protofibrils among other species [16, 268]. Major efforts have also been devoted to reducing the amount of aggregation-prone Aβ monomers by targeting the proteolytic cleavage of APP, its precursor protein, by secretases [382, 383]. This in theory would reduce the number of oligomers formed, but this strategy has demonstrated limited success in clinical trials.

### Reduction of oligomer interactions

In addition to inhibiting the formation of oligomers, promoting their clearance, changing their structure, and attenuating the aberrant interactions of the oligomers with their biological targets, another promising approach is the mitigation of specific oligomer-membrane protein interactions. This can be accomplished through targeting the action of oligomers on specific receptors, as exemplified by studies on microglial Aβ-induced P2X7R-dependent stimulation of inflammation and toxicity, which can be eliminated by the dihydropyridine nimodipine [384], and molecules that inhibited Aβ-LilrB2 interactions on the cell surface with reduced cytotoxicity [385]. Whether preventing the interaction of misfolded protein oligomers with cell membranes generally [366] or via specific receptor proteins in the membrane [385], these approaches aim at reducing the interaction of Aβ with the cell membrane, inhibit cell membrane destabilization and uptake of Aβ into the cytosol of the cell, which are known to lessen the toxicity of soluble Aβ species [386].

Several molecular chaperones have demonstrated capacity to modulate the size or hydrophobicity of misfolded protein oligomers. Sub-stoichiometric concentrations of ⍺B-crystallin, heat shock protein 70, clusterin, haptoglobin, and ⍺2-macroglobulin reduced the toxicity of Aβ_42_, IAPP, and HypF-N oligomers by promoting their assembly into markedly larger species with reduced diffusional mobility and reactive surfaces of the oligomers [308]. On the other hand, super-stoichiometric concentrations of the same chaperones can bind to the hydrophobic surfaces of the oligomers and neutralize them in the absence of their further clustering [387]. Heat shock protein B1 and transthyretin have also been characterized to sequester Aβ_42_ oligomers into inert species [388–391]. Clusterin has been shown to bind to hydrophobic portions of Aβ_42_ oligomers, therein slowing down its aggregation by inhibiting primary and secondary nucleation [392]. Of note, small molecules and protein engineering have been used to stimulate or attenuate the activity of specific molecular chaperones as therapeutic means [381].

It has also been reported that cell membranes were protected from the deleterious effects of misfolded protein oligomers using compounds that do not bind to oligomers or impact their structures, but rather integrate directly into the cell membrane [366]. Key aminosterols such as squalamine and trodusquemine strongly prevent and displace the binding of oligomers of ⍺-synuclein, Aβ_40_, Aβ_42_, and HypF-N to cell membranes, resulting in the attenuation of their toxicity to cultured cells [257, 258, 332, 362, 393], and in transgenic *C. elegans* models of PD [332, 362] and AD [257] diseases. These molecules additionally have effects on the kinetics of amyloid formation and therefore also impact the rate of oligomer formation [257, 332, 362]. Similarly, one report found that EGCG caused a partial reduction in the binding of ⍺-synuclein oligomers to vesicles and cells without impacting the secondary structure or size of the isolated oligomers [394]. Modulating the lipid composition of the neuronal membrane with endogenous factors such as GM1 and cholesterol can also mitigate oligomer binding and their associated toxicity [306, 314]. Collectively, these results highlight the importance of physicochemical properties for both misfolded protein oligomers and plasma membranes in mediating the binding and ultimate toxicity of oligomeric aggregates.

It is also possible to target the deleterious immunological effects of misfolded protein oligomer toxicity. Elevated markers of brain inflammation such as interleukin (IL)-1β, tumor necrosis factor (TNF), and IL-6 have been found in the CSF, brains, and serum of patients with various neurodegenerative diseases [395, 396]. Recent work demonstrated that IL-1β regulates the dysfunction induced by Aβ oligomers to mitochondrial proteins [397], and blocking TNF receptor-1 genetically or pharmacologically was beneficial in APP/PS1 transgenic mice [398]. The molecule nimodipine has also been shown to reduce IL-1β levels caused by intra-hippocampal inoculation with Aβ [384]. 

**Fig. 10:**
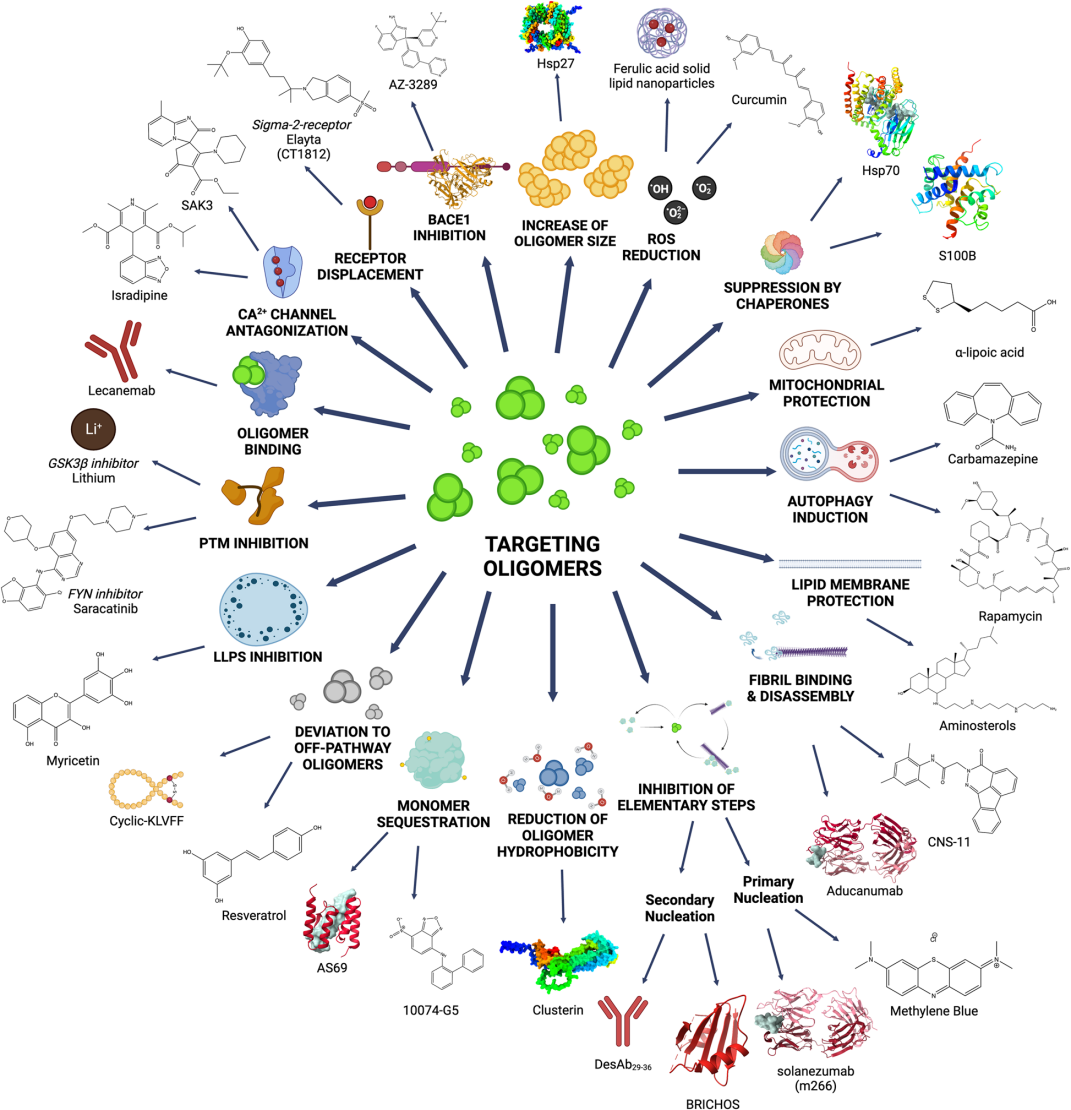
Overview of approaches under development that could be potentially able to suppress the formation, reduce the lifetime, or decrease the toxicity of misfolded protein oligomers. Biological or chemical structures of prototypical drugs are shown for each class of therapeutic. Created with biorender.com

An overview of a subset of the different classes of therapeutics being developed to target oligomers is summarized in Fig. 10, including seminal molecules corresponding to each approach [101, 179, 272, 371, 378, 388, 392, 399–430].

## Conclusions

In order to provide a molecular-level understanding of current therapeutic strategies that are being explored for the treatment of protein misfolding diseases, and to inspire new ones, we have described the mechanisms by which misfolded protein oligomers form, interact with cells and induce cytotoxicity, as well as approaches investigated to mitigate their toxicity. Support to therapeutic strategies targeting misfolded protein oligomers, such as those discussed in this review, has come from the recent accelerated approval of the antibodies lecanemab and aducanumab by the FDA for the treatment of AD. These advances, however, should not confuse the fact that AD is a multifactorial disorder characterized not just by Aβ and tau aggregation, but also by excitotoxicity, synaptic loss, inflammation, cholinergic dysfunction, oxidative stress, glucose hypometabolism, alterations of the gut microbiome, the immune pathway, the endocrine pathway, and bacteria-derived metabolites [431]. Other neurodegenerative diseases such as PD are also multifactorial. On the misfolded protein oligomer side, further progress will require major developments in two areas. The first is the establishment of quantitative methods for the detection of oligomers in vivo and for investigating their mechanisms of formation. The second is the development of toxicity assays that recapitulate pathological mechanism relevant in disease. With continued research, we anticipate that more effective strategies to both achieve early diagnosis and develop compounds to target oligomeric species will lead to the generation of effective disease-modifying therapeutics for a wide variety of protein misfolding diseases.

## Data Availability

New data were not generated for this review.

## Abbreviations

3R: Three-repeat Tau
4R: Four-repeat Tau
Aβ: Amyloid-β peptide
AD: Alzheimer’s Disease
ADDLs: Amyloid-derived diffusible ligands
AFM: Atomic force microscopy
AMPA: α-Amino-3-hydroxyl-5-methyl-4-isoxazolepropionic acid
ANS: 8-Anilinonaphthalene-1-sulfonate
ASK1: Apoptosis signal-regulating kinase 1
ATTR: Amyloid transthyretin amyloidosis
BLI: Biolayer interferometry
CBD: Corticobasal degeneration
CD: Circular dichroism
Cryo-EM: Cryogenic electron microscopy
CSF: Cerebrospinal fluid
CTE: Chronic traumatic encephalopathy
DLS: Dynamic light scattering
EGCG: (-)-Epigallocatechin gallate
ELISA: Enzyme-linked immunosorbent assay
EMA: European Medicines Agency
FcγRIIb: Fcγ receptor IIb
FDA: U.S. Food and Drug Administration
FRET: Förster resonance energy transfer
FTIR: Fourier transform infrared spectroscopy
HD: Huntington’s disease
IAPP: Islet Amyloid Polypeptide (amylin)
IL: Interleukin
LilrB2: Leukocyte immunoglobin-like receptor B2
LRP1: Low-density lipoprotein receptor-related protein-1
MCI: Mild cognitive impairment
MSA: Multiple system atrophy
NMDA: N-methyl-D-aspartate
PD: Parkinson’s Disease
PICUP: Photo-induced cross-linking of unmodified protein
PiD: Pick’s disease
PrP: Prion protein
PSP: Progressive supranuclear palsy
ROS: Reactive oxygen species
RT-QuIC: Real-time quaking-induced conversion
SKAR: Structure-kinetic activity relationship
SPR: Surface plasmon resonance
ssNMR: Solid-state nuclear magnetic resonance
T2D: Type II Diabetes
TDP-43: TAR DNA-binding protein 43
TNF: Tumor necrosis factor
TTR: Transthyretin
